# Unravelling tumour spatiotemporal heterogeneity using spatial multimodal data

**DOI:** 10.1002/ctm2.70331

**Published:** 2025-05-07

**Authors:** Chunman Zuo, Junchao Zhu, Jiawei Zou, Luonan Chen

**Affiliations:** ^1^ School of Life Sciences Sun Yat‐sen University Guangzhou China; ^2^ Key Laboratory of Systems Biology, Shanghai Institute of Biochemistry and Cell Biology, Center for Excellence in Molecular Cell Science Chinese Academy of Sciences Shanghai China; ^3^ Key Laboratory of Systems Health Science of Zhejiang Province, School of Life Science, Hangzhou Institute for Advanced Study University of Chinese Academy of Sciences Chinese Academy of Sciences Hangzhou China; ^4^ West China Biomedical Big Data Center, Med‐X Center for Informatics West China Hospital Sichuan University Chengdu China; ^5^ School of Mathematical Sciences and School of AI Shanghai Jiao Tong University Shanghai China

**Keywords:** clinical diagnosis and treatment, spatial multimodal integration, tumour spatial and temporal heterogeneity

## Abstract

Analysing the genome, epigenome, transcriptome, proteome, and metabolome within the spatial context of cells has transformed our understanding of tumour spatiotemporal heterogeneity. Advances in spatial multi‐omics technologies now reveal complex molecular interactions shaping cellular behaviour and tissue dynamics. This review highlights key technologies and computational methods that have advanced spatial domain identification and their pseudo‐relations, as well as inference of intra‐ and inter‐cellular molecular networks that drive disease progression. We also discuss strategies to address major challenges, including data sparsity, high‐dimensionality, scalability, and heterogeneity. Furthermore, we outline how spatial multi‐omics enables novel insights into disease mechanisms, advancing precision medicine and informing targeted therapies.

**Key points:**

- Advancements in spatial multi‐omics facilitate our understanding of tumour spatiotemporal heterogeneity.
- AI‐driven multimodal models uncover complex molecular interactions that underlie cellular behaviours and tissue dynamics.
- Combining multi‐omics technologies and AI‐enabled bioinformatics tools helps predict critical disease stages, such as pre‐cancer, advancing precision medicine, and informing targeted therapeutic strategies.

## Graphical abstract

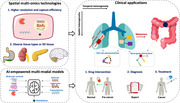

## 1 INTRODUCTION

The human body contains trillions of cells, encompassing a wide range of types and functional states. These cells are shaped by complex intra‐ and intercellular networks to form intricate tissue across organs and systems. Internally, dynamic interactions among nucleic acids, proteins, metabolites, and RNA influence cellular state.^1^ Externally, neighbouring cells impact cell behaviour through mechanisms like ligand–receptor interactions,^2^ and chemical gradients.^3^, ^4^ In healthy systems, these varied cell types work in coordination with time and space to maintain tissue stability and homeostasis. In contrast, in disease, disruptions frequently occur as a result of shifts in cell type composition and organisational patterns.^5^, ^6^ Elucidating how the structure and function of cells change over time and space is crucial for deciphering disease mechanisms. This is because the differences in molecular, cellular, and structural patterns usually reflect their roles and functions in the body.^7^

In this review, we explore current approaches for dissecting tumour spatiotemporal heterogeneity using spatially resolved omics technologies and related computational tools, with a particular focus on spatiotemporal models designed to capture spatial and temporal dependencies within data. We highlight recent studies showing major advancements in spatial multi‐omics technologies and their applications in cellular biology and clinical research. Our selection prioritises peer‐reviewed articles that offer insights into multimodal fusion, featuring translational applications in disease contexts. We acknowledge that due to space constraints, many important studies could not be included.

### 1.1 Deciphering tumour progression over time and space

Tumour development is shaped by genetic mutations and the makeup of nearby microenvironment cells.^8^, ^9^ The transformation from normal to cancerous cells includes accelerated growth, evasion of growth controls, initiation of new blood vessel formation, and activation of invasive and metastatic pathways.^10^ Cancer often arises through random events, underscoring the complex and adaptive nature of its progression. Consequently, different tumours display a variety of molecular differences, including genetic mutations, changes in gene expression (transcriptomics), DNA modifications that affect gene activity (epigenetics), and visible changes in cells (phenotypic changes).^11^, ^12^

Tumour heterogeneity encompasses genetic and phenotypic differences within and between tumours. It can be divided into two main categories: inter‐ and intra‐tumoural heterogeneity. The former refers to variations across tumours from different patients, influenced by factors such as genetic mutations and environmental factors. The latter pertains to differences within a single tumour, which can be spatial (across distinct regions) or temporal (over time). Spatial heterogeneity involves the presence of genetically diverse populations within various tumour regions, while temporal heterogeneity captures changes in the tumour's genetic profile over time.^11^, ^13^ Research indicates that intra‐tumoural heterogeneity is a key driver of cancer progression and resistance to treatment.^14^ Therefore, understanding these spatiotemporal variations is crucial for developing targeted and sustainable therapeutic approaches.

### 1.2 Spatial multi‐omics technologies and computational methods

#### 1.2.1 Spatial multi‐omics technologies

**FIGURE 1:**
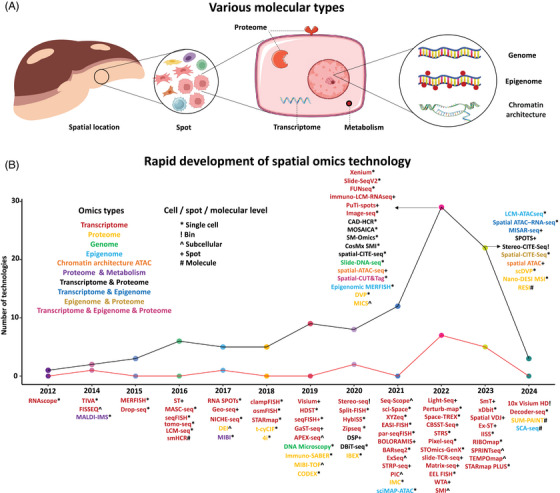
Spatial multi‐omics sequencing techniques. (a) Overview of sequencing technologies used to characterise diverse molecular features for studying cellular heterogeneities, including spatial transcriptomics, proteomics, chromatin openness, protein expression, genomics, and epigenomics. (b) Timeline showing the development of spatial omics technologies, where different colours indicate various molecular features, and distinct symbols represent different levels of resolution. Note: ‘*’ denotes single‐cell resolution, ‘!’ denotes bin resolution, ‘^’ indicates subcellular resolution, ‘+’ represents spot indicates spot‐level resolution, and ‘#’ signifies molecular‐level resolution. The upper line depicts the cumulative number of spatial omics technologies developed over time, while the lower lines specifically track the rise of multi‐omics technologies.

Spatial omics technologies allow the simultaneous measurement of diverse molecular features – such as the genome,^15^, ^16^ epigenome,^17^, ^18^, ^19^, ^20^, ^21^, ^22^, ^23^, ^24^, ^25^, ^26^ transcriptome,^27^, ^28^, ^29^, ^30^, ^31^, ^32^, ^33^, ^34^, ^35^, ^36^, ^37^, ^38^, ^39^, ^40^, ^41^, ^42^, ^43^, ^44^, ^45^, ^46^, ^47^, ^48^, ^49^, ^50^, ^51^, ^52^ proteome,^53^, ^54^, ^55^, ^56^, ^57^, ^58^, ^59^, ^60^, ^61^, ^62^, ^63^, ^64^, ^65^, ^66^, ^67^, ^68^, ^69^, ^70^, ^71^, ^72^, ^73^ and metabolome^74^, ^75^ while maintaining their spatial information^76^, ^77^ (Figure 1 and Table S1). These technologies have significantly advanced our ability to explore molecular, cellular, and structural patterns in both healthy and diseased states. Highlighted by Nature in 2022^78^ as a key technology, spatial multi‐omics has developed from previous spatial mono‐omics methods.^70^, ^79^, ^80^, ^81^, ^82^, ^83^, ^84^, ^85^ Innovations like MISAR‐seq enable combined chromatin accessibility and transcriptome analysis,^85^ while SPOTS supports simultaneous proteomics and transcriptomics profiling.^83^

Spatially resolved transcriptomics (SRT) data has emerged as a popular method for analysing disease progression, particularly within tumours,^86^, ^87^ as it allows for quantifying gene expression while maintaining spatial information within tissues. SRT methods are divided into imaging‐based and sequencing‐based approaches. In imaging‐based methods, single‐molecule fluorescent in situ hybridisation (smFISH)^88^ quantifies multiple mRNA transcripts at subcellular resolution, with subsequent methods like seqFISH,^89^ seqFISH+,^30^ and MERFISH^90^ multiplexed capabilities. Recently, nanoString commercialised the CosMx™ SMI platform to provide spatial multi‐omics with FF and FFPE tissue samples at cellular resolution, quantifying up to 6000 RNAs and 64 proteins.^91^ Sequencing‐based techniques, such as ST,^92^ MASC‐seq,^93^ Slide‐seq,^94^ Slide‐seqV2,^95^ and HDST,^35^ measure the expression across spatial spots. 10x Genomics's Visium platform^96^ has improved its resolution, reducing spot diameter from 100 to 55 µm. Furthermore, the recently released Visium HD enables resolutions of 2, 8, and 16 µm, while the Xenium platform offers true single‐cell subcellular resolution. BGI's Stereo‐seq achieves 500nm resolution over larger tissue areas.^97^ The sequencing‐based technologies yield multimodal data, including gene expression, spatial location, and histology, the integration of which helps to reveal complex tissue architecture.^5^

#### 1.2.2 Computational strategies

**FIGURE 2:**
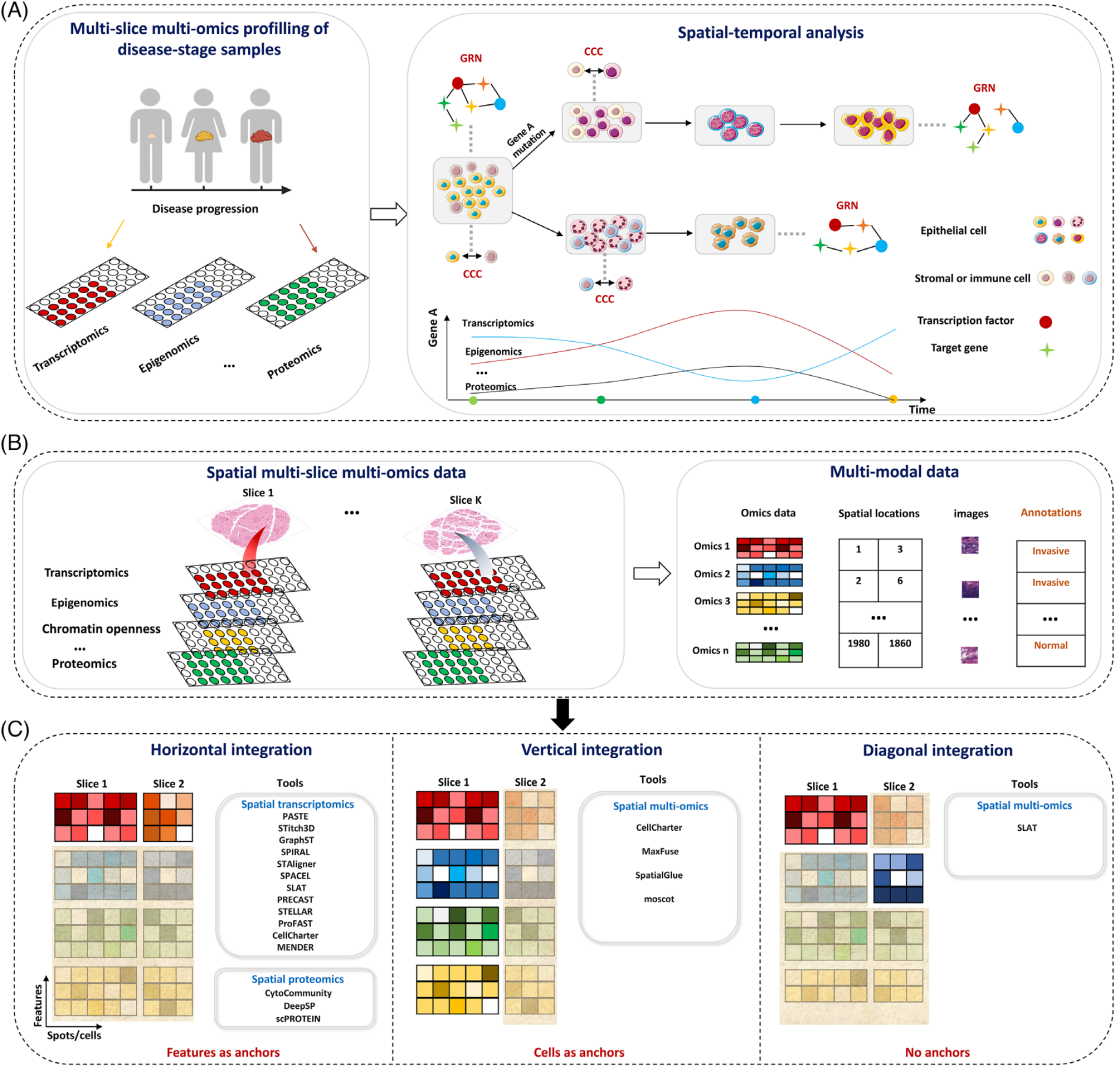
Overview of key spatial multi‐omics integrative analysis methods and their application. (a) Integrating spatial multi‐slice multi‐omics data across multiple time points during disease progression, enables a detailed analysis of tumour spatiotemporal heterogeneity and facilitates the identification of intra‐ and intercellular molecular relationships within the context of underlying genetic background, as well as the characterisation of omics‐level variations along pseudo‐time. GRN: gene regulatory network; CCC: cell–cell communication. (b) Various molecular technologies applied to two tissue slices produce multimodal data, encompassing omics data (e.g., transcriptomics, and proteomics), spatial coordinates, imaging data, and possibly histological annotations. (c) Spatial multi‐omics data integration can be categorised into three types: horizontal, vertical, and diagonal integration, defined by specific anchors (i.e., reference cells/spots) and features. Horizontal integration involves combining the same omics data type across multiple slices to achieve a broader spatial context, vertical integration aligns and integrates multiple omics layers within a single slice, providing a multidimensional view of molecular interactions in a localised area. Diagonal integration bridges different omics layers and different slices, enabling cross‐sectional comparisons and insights into molecular heterogeneity between tissue regions.

Integrating diverse spatial multi‐slice multi‐omics data facilitates characterising dynamic behaviours of different types of molecules, intracellular molecular networks, and intercellular regulation in the spatiotemporal progression of tumours (Figure 2a). However, spatial omics technologies often face resolution and capture efficiency challenges. Enhancing sensitivity and specificity by integrating public single‐cell omics data or known interactions is thus crucial in bioinformatics. In this section, we will discuss key integration challenges, strategies for effective integration, current limitations and future directions.

The tools are divided into three categories based on how reference cells/spots are selected (Figure 2b and c and Table S2):^98^ specifically, (i) for identical omics types across slices (horizontal integration), shared features across these slices serve as reference points; (ii) for different types of omics data from the same tissue slice (vertical integration), such as when both gene expression and protein data are collected using DBiT‐seq technology,^79^ the individual cells serve as the reference; and (iii) when different types of omics data are obtained from different tissue slices (diagonal integration), no common reference exists because the data types do not share similar features. For instance, gene expression looks at how genes are activated, while genetic data measures mutation throughout the genome. This difference in data types presents an initial challenge for combining these multi‐omics data. In the following sections, we will explain the methods and analysis strategies for overcoming these practical challenges when integrating spatial multi‐omics data.

In the analysis of omics data from multiple slices, computational methods are typically divided into two categories: those that deal with slices from the same tissue and those from different tissues. Integrating multi‐slices omics data presents several challenges: (i) variations in tissue composition that affect cell densities, structures, and the surrounding microenvironment; (ii) physical shifts or distortions that make it difficult to align slices correctly; (iii) batch effects due to differences in how the samples were prepared, which can mask true biological signals; (iv) inconsistent markers leading to information gaps; (v) differences in resolution and detecting methods; and (vi) the risk of amplifying poor‐quality data or noise. When slices come from different tissues, an additional layer of biological variation must be considered. Addressing these challenges requires advanced alignment algorithms, batch correction techniques, and noise reduction methods to ensure accurate integration and interpretation. Here, we illustrate strategies for addressing these challenges, using the integration of multi‐slice SRT data as a representative example. Specifically,

(1) For multiple sections from the same tissue, PASTE^99^ applies the optimal transport (OT) method to map and analyse neighbouring slices. Building on this, STitch3D^100^ and GraphST^101^ use PASTE (or iterative closet point algorithm) to create a unified graph with 3D spatial coordinates and then apply a graph model to learn embeddings for spatial clustering. However, linear alignment in PASTE and its derivatives has limitations in detecting distortions in complex structures within slices caused by diseases with high variability. Moreover, SPACEL^102^ leverages graph models to predict cell type proportions, identify spatial domains, and reconstruct 3D tissue structure.

(2) For multiple slices across tissues: SEDR^103^ combines gene expression and spatial coordinates using an autoencoder and graph model for spatial clustering. PRECAST^104^ performs dimension reduction and spatial clustering via projection‐based alignment, and its latest version, FAST,^105^ is designed for large‐scale data across slices. STAligner^106^ uses a graph model and spot triplets to identify shared and conditional clusters. SLAT^107^ employs graph and adversarial learning algorithms to map slices across technologies/omics. SPIRAL^108^ integrates graph and OT methods to remove batch effects, predict unseen samples, and align coordinates. STELLAR^109^ uses graph geometric learning via cell representations to transfer annotations from one slice to another across regions, tissues, and donors. However, these methods do not fully leverage the intricate inter‐spot relations within and across slices, limiting their ability to capture partial relations in heterogeneous slices.

While most methods focus on integrating omics data with spatial location, they often overlook the complementary insights from modalities like histological images and annotations. Effectively leveraging this additional information – while addressing challenges related to scale, diversity, multimodality, and high dimensionality (where each sample contains a large number of features) – remains a complex task. Another important challenge we would like to highlight is how to utilise the large populations of cells/spots in tissue slices and phenotypes in tissue slices to obtain phenotypically relevant biological findings. Recently, CytoCommunity,^110^ DeepSP,^111^ and scPROTEIN^112^ have been introduced to integrate spatial proteomics data, offering new approaches for handling spatial information across multimodalities. For other omics data – such as chromatin openness and metabolism^113^, ^114^ across multi‐slices – these methods provide useful frameworks for inspiration.

In integrative analysis of spatial multi‐omics data from the same slices, most methods address the challenges such as (i) differences in scale and measurement units across omics types; (ii) sparsity, low resolution, and noise in multi‐omics data; and (iii) high dimensionality, which requires substantial computational power. These methods typically begin by mapping data into a shared or coordinated feature space to minimise differences across omics. While single‐cell methods^115^, ^116^, ^117^, ^118^, ^119^, ^120^, ^121^, ^122^, ^123^ like scGPT, GLUE, totalVI, MultiVI, and Seurat (see previous review for details^124^) can be adapted for spatial data integration, they may not fully capture the spatial context information, which is important for elucidating tissue composition. Combining spatial coordinates with multi‐omics data is a new research area, and few methods have been proposed so far. For instance, Cellcharter^125^ and SLAT^107^ use preprocessing tools like scVI^126^ and GLUE,^115^ and then leverage graph models to learn cell/spot representations. MaxFuse^127^ smooths input data using graphs and iteratively maps different omics after co‐embedding. SpatialGlue^128^ combines spatial information with feature graphs using graph and attention methods, while moscot^129^ models cell mapping across time and space as an OT problem. PRESENT^130^ uses contrastive learning to capture cross‐modal representations in multi‐omics data. Moreover, for slices from different tissues, additional challenges arise due to biological heterogeneity and variations in resolutions that capture cellular features at different scales, complicating accurate alignment for integrative analysis.

Beyond the methods described for identifying spatial domains or cellular niches from batch‐corrected features through the integration of multi‐slice multi‐omics data, future research should focus on exploring potential conversion relationships between cellular niches and the intracellular and intercellular molecular interactions or regulations in driving disease progression. This can be achieved by integrating spatial locations with multi‐omics data,^131^ histological images, and annotations, as well as public single‐cell omics data and known molecular interactions.^132^ To improve the biological explanation and interpretation of spatial multi‐omics data, computational solutions can be developed in several key directions: (i) Fine‐tuning pre‐trained models derived from large‐scale single‐cell reference data to adapt to spatial omics data. This approach can enhance sparse or low‐resolution spatial data, enabling more precise cell type annotation and deeper function insights; (ii) leveraging known gene‐gene interactions or public ATAC‐seq profiles^133^ to construct associations across different molecular data types, facilitating the creation of cross‐modal relations in the context of disease proregression and mitigating the impact of low‐quality of omics data; and (iii) inferring cause relationships between regulator elements and target genes involved in disease progression, aiding in the identification of potential targets for therapeutic intervention. Such comprehensive integration would provide deeper insights into cellular interactions and the spatiotemporal evolution of disease, effectively addressing the inherent complexity and multidimensionality nature of spatial omics data.

### 1.3 SRT computational analysis

**FIGURE 3:**
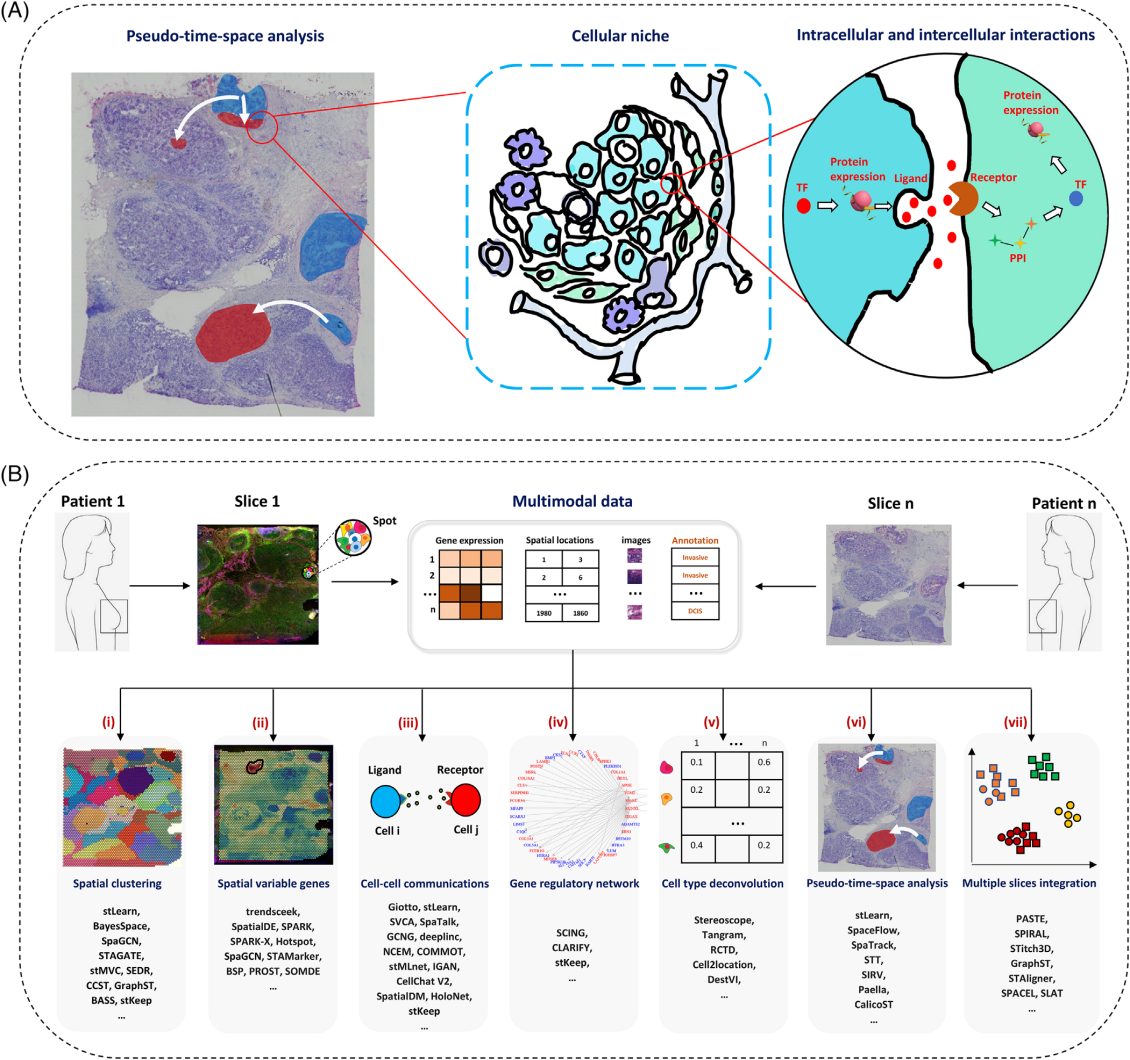
SRT multimodal data and its applications. (a) Integrative analysis of SRT multimodal data enables pseudo‐time‐space analysis and the examination of intracellular and intercellular interactions. TF: transcription factor, and PPI: protein–protein interaction. (b) SRT technology typically produces three types of multimodal data: gene expression, spatial locations, and Haematoxylin and Eosin (H&E) or immunofluorescence (IF) staining images. Pathologists can also provide pathological annotations for tumour slices. Common applications of SRT multimodal data integration include (i) spatial clustering to identify distinct tissue regions or structures; (ii) identification of SVGs, which show differentially expressed across spatial locations; (iii) inference of CCC to study interactions between neighbouring cells; (iv) prediction of GRN to map molecular interactions; (v) cell type deconvolution for the spot‐level SRT data, where the number of cells per spot depends on the spot's diameter; (vi) pseudo‐time‐space analysis to explore temporal changes in cellular states; and (vii) integration across multiple tissue slices for a comprehensive view of tissue heterogeneity.

Computationally integration of multimodality in SRT data can be used to accurately characterise regulatory and interaction networks within cells and with surrounding cells via secreted proteins during the spatiotemporal progression of the disease (Figure 3a). Here, we describe these methods and analysis strategies, addressing practical challenges in analysing spatial omics data (Figure 3b and Table S3), covering aspects: spatial clustering, detection of spatially variable gene (SVG), inference of cell–cell communication (CCC), prediction of gene regulatory network (GRN), cell type deconvolution of spot‐level SRT data, pseudo‐time‐space analysis, multiple slices integration or 3D reconstruction (see Section 1.2).

#### 1.3.1 Spatial clustering

In the analysis of SRT data to identify spatial domains within tissues, various methodologies have emerged as indispensable tools. Key statistical methods in the field, such as BayesSpace,^134^ Giotto,^135^ and DR‐SC,^136^ leverage probabilistic models to identify spatial components by combining gene expression and spatial coordinates. In contrast, deep learning models like SpaGCN,^137^ STAGATE,^138^ stMVC,^5^ and stKeep^139^ apply graph‐based approaches to reveal spatial patterns within multimodal data. A recent review provides detailed benchmark comparisons of these methods on simulated and real data.^140^

Despite recent progress, several challenges remain in the field. With the increasing availability of single‐cell and subcellular resolution data, addressing issues such as sparsity, high‐dimensionality, scalability, and interpretability is crucial for further progress. Overcoming these challenges is essential to fully unlock the potential of SRT data and to gain a deeper understanding of spatial tissue organisation and cellular composition.

#### 1.3.2 Identification of SVG

Detection of SVGs is essential for elucidating tissue biology, which involves identifying genes with differential expression across tissue or specific domains. Researchers have developed many computational approaches to address this problem, each with its strengths. For example, trendsceek^141^ uses a permutation process to estimate spatial dependencies, while SpatialDE^142^ applies Gaussian process regression to assess spatial variance. SPARK^143^ and SPARK‐X^144^ detect spatial patterns through statistical methods, SOMDE utilises self‐organising maps^145^ and Hotspot^146^ employs graph models. SpaGCN^137^ tests the hypothesis for each gene based on identified domains, and STAMarker^147^ uses saliency maps to highlight important features. BSP^148^ and its enhanced version scBSP^149^ use a big‐small patch algorithm to detect SVGs at different scales, while PROST^150^ introduces an indicator (PI) to evaluate spatial expression variation. A comparison of these methods is available in a recent review.^151^

Despite these advancements, challenges remain. Handling the high‐dimensional and spatially structured nature of the data, while ensuring robustness and scalability across diverse conditions, is difficult. Additionally, dealing with noise, tissue heterogeneity, and complex interactions within the tissue microenvironment, along with ensuring scalability to large or 3D data, is vital for detecting SVGs. Addressing these challenges will be key to dissecting gene expression patterns and functions of different spatial components in disease progression.

#### 1.3.3 Inference of cell–cell communications

Multicellular organism complexity arises from communication between various cell types. Computational inference of CCC from local spatial components is important for understanding cellular heterogeneity and functions,^2^ and can be divided into two categories: (1) cell‐population‐level: Giotto^135^ calculates interactions between neighbouring cell types based on ligand–receptor pairs. stLearn^152^ tests significant enrichment of ligand–receptor pairs in neighbouring cells using co‐expression analysis. CellPhoneDB v3^153^ examines interactions from the same local domains. SVCA^154^ and MISTy^155^ leverage statistical and machine‐learning models to infer spatially dependent cellular gene‐gene interactions. deeplinc^156^ constructs CCC maps from scratch based on ligand–receptor genes; and (2) single‐cell‐level: SpaTalk^157^ constructs and quantifies the ligand–receptor‐target signalling network between nearby cells through knowledge‐based graph models. NCEM^158^ calculates how the composition of the cellular environment affects gene expression using graph models. COMMOT^159^ leverages collective OT to infer CCC by considering the competition between different ligands and receptors and their spatial arrangement. stKeep^139^ adopts a heterogeneous graph (HG) to infer CCC, ensuring that learned CCC patterns are comparable across different cell states through contrastive learning. IGAN^160^ infers gene programs influenced by CCC using spatial correlation. A comparative analyses of some of these methods are provided in the recent review.^161^

Despite these advances, current methods still face significant challenges. Many struggle to capture the dynamic and context‐dependent nature of CCCs, especially in heterogeneous conditions and environments. Scalability and computational efficiency are also issues, partially when dealing with large‐scale datasets and integrating multi‐slice data. Advances in single‐cell and imaging technologies will be crucial for providing detailed insights into CCCs at both the cellular and molecular levels, improving our understanding of how cells communicate. In the context of spot‐level SRT data analysis (involving multiple cells), it is critical to dissect how various cells coordinately respond to dynamic conditions.

#### 1.3.4 Prediction of gene‐regulatory network

Cell identity is controlled by GRNs, and transcription factors (TFs) interact with enhancers and promoters to regulate gene expression. Inferring GRNs from omics data is key to identifying impaired gene functions and critical drivers of disease progression. Accurately inferring regulatory networks that characterise cell states while addressing challenges such as high dimensionality, sparsity, and high noise in omics data is very challenging, especially for the spot‐level (multiple cells) data. As a result, there are currently few research methods available. For example, SCING^162^ utilises gradient boosting and mutual information methods to infer stable GRNs. CLARIFY^163^ employs a graph model to construct cellular networks, supporting CCC inference and enhancing the accuracy of cell‐specific GRNs. stKeep^139^ leverages an attention‐based multi‐relation graph embedding method to aggregate information from cells and cell states while ensuring that co‐related genes are co‐embedded to learn gene embeddings. The embeddings can be used to identify cell‐state‐specific GRNs.

We want to highlight that it's important to be cautious when interpreting results from spot‐level omics data. That is because the relations between two genes in these omics do not always indicate they are co‐regulated or co‐expressed within a cell. stKeep solves this by using known relations between genes from public databases to avoid false positives. However, some co‐associated gene pairs within one cluster may still be missed. Moreover, it is important to infer the direction of gene regulation in a cell. Leveraging spatial multi‐omics data, along with unpaired ATAC‐seq or Chip‐seq data, is essential for uncovering gene‐gene relations and their directions.^131^

#### 1.3.5 Pseudo‐time‐space analysis

Pseudo‐time‐space methods allow researchers to track cell state changes throughout tissue space and time, providing insights into homeostasis, repair, and responses to environmental signalling.^164^ To explore temporal changes within SRT data, several methods have been developed. SpaceFlow^165^ leverages graph model to learn cell/spot features through combining gene expression and spatial coordinates then calculates pseudo‐spatiotemporal MAP (pSM) using a diffusion pseudo‐time (DPT) algorithm.^166^ stLearn,^152^ STAGATE, and stMVC construct a spatial PAGA graph using gene expression similarity between spatial domains, and infers the pseudo‐temporal order among these domains using a minimum spanning tree algorithm. SIRV^167^ estimates RNA velocities at single‐cell resolution by incorporating spliced and un‐spliced mRNA data from reference scRNA‐seq into SRT. Paella^168^ uses initial pseudo‐temporal values and spatial coordinates to create a network that progressively identifies several sub‐trajectories. STT^169^ uses a dynamic model to describe multistability in space through mRNA splicing and SRT data. spaTrack^170^ creates spatial pseudo‐temporal sequences by addressing OT problems between two cell groups. Additionally, spatial RNA velocity provides a way to directly infer developmental trajectories by representing temporal changes in cells.^171^ Recently, CalicoST^172^ has enabled the simultaneous inference of allele‐specific copy number aberrations while also reconstructing spatial tumour evolution from SRT data.

In the future, more efforts should focus on identifying key regulators and genes that drive the spatial and temporal transitions of 3D tissue space to better understand cell differentiation and disease progression. Moreover, enhanced methods to fill in missing temporal data will be important to study dynamic cellular behaviours and increase the data's usefulness.

#### 1.3.6 Cell type deconvolution

High‐throughput platforms such as Visium capture the full transcriptome but lack single‐cell resolution. Moreover, tissue slice thickness can also cause overlapping RNA signals from multiple cells. Imaging‐based SRT methods achieve subcellular resolution but are limited in gene numbers, restricting their broader use. Hence, accurate prediction of cell types in spot‐level SRT data is crucial for identifying disease‐associated cellular composition and structures.

Current integrative analysis of whole‐transcriptome and scRNA‐seq data falls into two categories: (1) deconvolution‐based methods: CARD,^173^ Cell2location,^174^ RCTD,^175^ and POLARIS,^176^ which use statistical or probabilistic‐based models to spatial map cell types. DestVI^177^ utilises deep learning to capture gene expression differences among cells of identical type. GraphST^101^ and CellMirror^178^ use contrastive learning to estimate cell type proportions. DSTG^179^ and STdGCN^180^ leverage graph models to predict cell type composition based on graphs created from both real and simulated SRT data, with the simulated data generated from the single‐cell reference database. Redeconve^181^ estimates the single‐cell composition of SRT spots through non‐negative least regression method; (2) alignment‐based methods: NovospaRc,^182^ Tangram,^183^ Celltrek,^184^ and CytoSPACE,^185^ map single‐cell locations to SRT data by analysing gene expression similarities; and (3) reference‐free methods like Stdecon^186^ and RETROFIT^187^ handle challenges between scRNA‐seq and SRT data, including batch‐effects, uneven of cell type coverage, and variations in gene expression.

Most current methods provide the proportion of different cell types in each spot, but do not provide the precise localisation of each cell type within the spot, indicating a need for computationally improved resolution in the future. Integrating histological images as complementary information could help enhance this resolution. In addition, when using deconvolution results for further CCC inference, caution is needed, as many different cell types may express the same ligand.

### 1.4 Integrating large‐scale public omics and imaging data

Computational biology and pathology are undergoing major changes,^122^, ^188^, ^189^ with the rapid development of artificial intelligence (AI) research and the public availability of various omics and imaging data. For example, some transformer‐based AI models like scGPT,^122^ scBERT,^190^ Geneformer,^191^ and scFoundation^192^ are designed to combine and analyse large amounts of single‐cell omics or multi‐omics data. These tools mainly encode cells from gene expression data, but face challenges in analysing spatial omics data because they do not use spatial location information. Moreover, histological imaging, which is essential for characterising tissue structure and disease status at a microscopic level, should be integrated with gene expression data to provide a clearer picture of disease development across time and space.

Decoding gene expression from histological images is crucial for understanding tissue structure and development, while also avoiding the need for additional sequencing costs. Current methods often involve transforming image patches into simplified representations, encoding these into features, and then decoding them to predict gene expression profiles. Image patches are processed using techniques like CNNs or transformers (e.g., Hist2RNA,^193^ Hist2ST,^194^ tRNAsformer,^195^ TCGN,^196^ BrST‐Net,^197^ and ST‐Net^198^) or simpler linear encoders (HisToGene,^199^ SEPAL,^200^ and HE2RNA^201^). Some methods, like TCGN,^202^ SEPAL,^200^ and IGI‐DL,^203^ utilise graph models to improve the embedding. In addition, BLEEP^204^ leverages the contrastive learning method to learn shared features for the alignment of images and gene expression data, helping find the closest reference expression profiles for new histology images. As spatial multi‐omics technology advances, future studies may integrate imaging data with genomic, transcriptomic, and proteomic data to better predict and diagnose disease.

### 1.5 Clinical applications

The integration of spatial multi‐omics holds significant promise for clinical translation, particularly in tumour diagnosis, prognosis, and treatment stratification. For example, SRT data has been used to identify immunosuppressive niches in breast cancer, enabling the discovery of localised treatment strategies.^5^, ^205^, ^206^ In liver disease research,^207^ the integration of single‐cell RNA‐seq with MERFISH technology has provided insights into the cellular architecture and spatial signalling patterns that drive disease progression. In our previous study,^139^ we developed heterogeneous graph model, stKeep, to infer GRN and CCC from spatial multimodal data. By applying stKeep to primary colorectal cancer and matched liver metastasis samples, we identified a key CCC axis – EREG/AREG→ERBB3 – that may mediate metastatic colonisation in liver tissue. These examples demonstrate the potential of spatial multi‐omics to uncover clinically related molecular interactions and guide precision medicine strategies.

### 1.6 Perspectives

**FIGURE 4:**
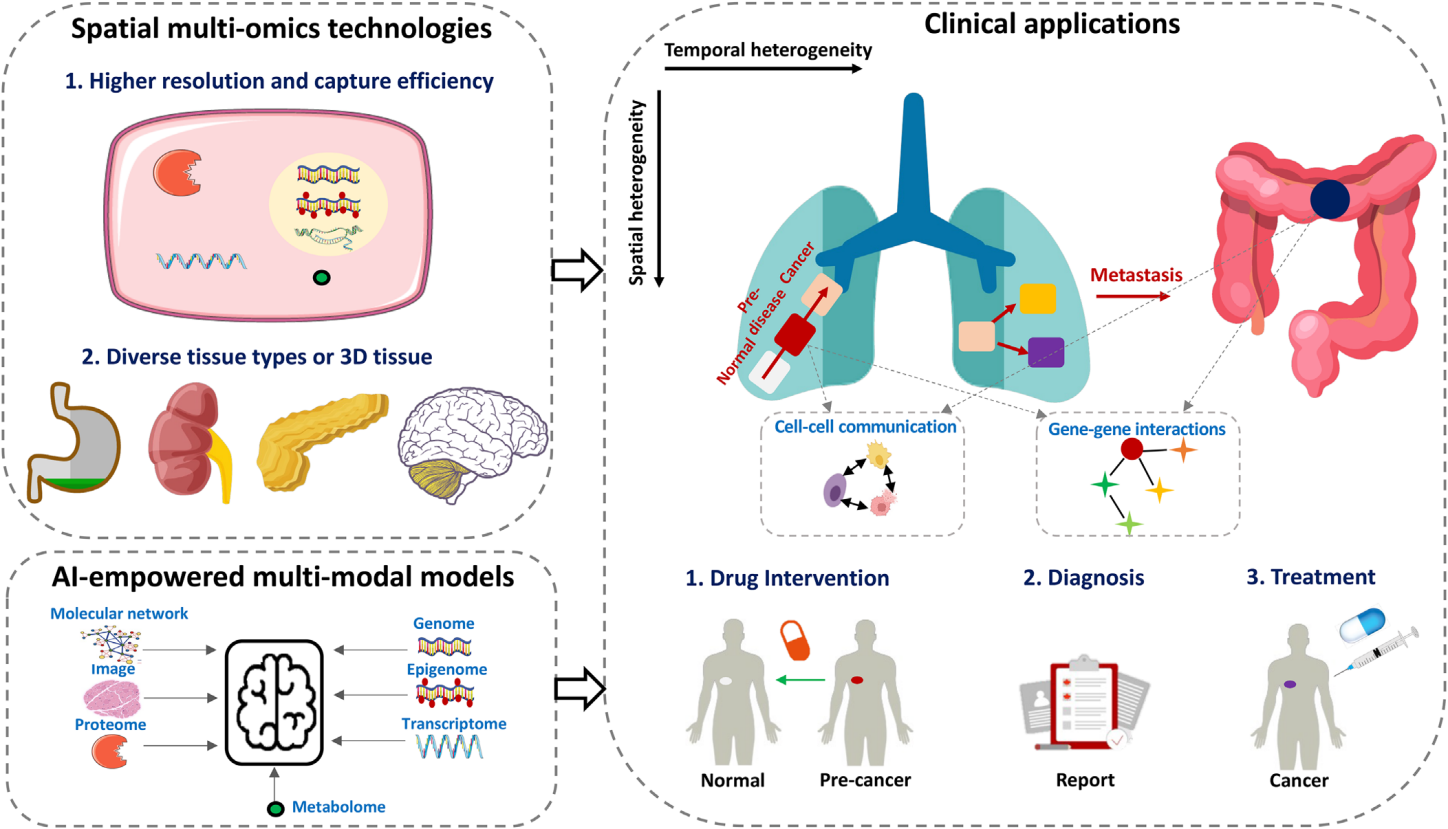
Future perspectives. Advancing spatial multi‐omics technologies to enhance resolution and data capture, developing artificial intelligence (AI)‐driven integrative tools for more robust multimodal data analysis, and expanding clinical applications to harness spatial multi‐omics in diagnostics, prognostics, and personalised treatment strategies.

Although the combination of AI and spatial omics technologies has driven the development of biomedical research in the past decade, and has great potential, there are still some areas that need improvement. These include enhancing omics technologies, developing AI‐driven bioinformatics tools, and advancing clinical applications (Figure 4): specifically, (1) understanding spatiotemporal evolution of cells needs high‐resolution, efficient quantification of multiple molecular features within single cells in a spatial context. Such methods are crucial for uncovering the underlying mechanisms and patterns of disease progression. They can be widely applied to analyse diverse tissue types and hold even greater potential when applied to resolve 3D tissue; (2) the development of multimodal AI methods^208^ helps integrate various data types, including image, various omics, and molecular networks (e.g., protein–protein interactions, gene regulatory networks, and ligand–receptor interactions). This integration aids in elucidating how cell systems regulate themselves and coordinate with surrounding cells to adapt to the external environment. Additionally, the use of foundation models trained on large corpora, images, or in the domains of medical imaging and single‐cell analysis opens new avenues for knowledge transfer and fine‐tuning for specific tasks. Such data integration and mining will contribute to a more comprehensive understanding of the spatiotemporal heterogeneity of diseases; and (3) predicting critical stages in the spatiotemporal progression of a disease, such as the pre‐disease or pro‐metastasis,^209^, ^210^ and identifying crucial factors driving transitions could provide key targets for clinical intervention, diagnosis, and treatment.

Together, these advancements, along with publicly available omics profiles for various diseases and the continued development of computational tools, will be crucial in dissecting cellular heterogeneity and spatiotemporal progression of diseases. This comprehensive approach provides a foundation for identifying critical transitions – such as tipping points preceding early cancer – and for predicting key driver factors that trigger these transitions.^6^, ^209^ Such insights may inform early warning strategies and enables targeted interventions to prevent cancer initiation.

## AUTHOR CONTRIBUTIONS

C.M.Z. and L.N.C. conceived the review. C.M.Z. wrote the manuscript with feedback from all authors. C.M.Z., J.C.Z., and J.W.Z. collected the materials. All authors contributed to the discussions. The authors read and approved the final manuscript.

## CONFLICT OF INTEREST STATEMENT

The authors declare no competing interests.

## ETHICS STATEMENT

Data sharing not applicable to this article as no datasets were generated or analysed during the current study.

## Supporting information



Supporting Information

Supporting Information

Supporting Information
