# Supplementary material for: Unravelling tumour spatiotemporal heterogeneity using spatial multimodal data
Source: Clin Transl Med. 2025 May 7;15(5):e70331. doi: 10.1002/ctm2.70331 (PMC12059211; doi:10.1002/ctm2.70331)
Supplement: Supplementary file 1 — Supporting Information [file CTM2-15-e70331-s003.docx]

| **Name** | **Type** | **Resolution** | **Year** | **Tissue/source** | **#Feature** | **# Cell/spot** | **Technology** | **Ref** |
| --- | --- | --- | --- | --- | --- | --- | --- | --- |
| RNAscope | Transcriptome | Single cell | 2012 | Human breast, liver, cervix cancer cells | several | ~100 | in situ hybridization | ^1^ |
| TIVA |  | Single cell | 2014 | Human brain, mouse brain | ~9000 | 1 | spatial labeling | ^2^ |
| MERFISH |  | Single cell | 2015 | Human fibroblast cells | 100~1000 | ~100 | in situ hybridization | ^3^ |
|  |  | Single cell | 2016 | Human osteosarcoma cells | 130 | ~40000 | in situ hybridization | ^4^ |
|  |  | Single cell | 2016 | Mouse Brain | 130 | >10000 | in situ hybridization | ^5^ |
|  |  | Single cell | 2018 | Mouse hypothalamic preoptic region | 135 | ~31000 | in situ hybridization | ^6^ |
|  |  | Single cell | 2019 | human osteosarcoma cells | ~10000 | 645 | in situ hybridization | ^7^ |
| FISSEQ |  | Single cell | 2014 | Human primary fibroblast | ~8000 | hundreds | in situ sequencing | ^8,9^ |
| Drop-seq |  | Single cell | 2015 | Mixtures of human HEK and mouse 3T3 cells, mouse retinal cells | ~12000 | thousands | barcode | ^10^ |
| ST |  | Spot | 2016 | Mouse olfactory  bulb | ~5000 | ~280 | barcode | ^11^ |
| MASC-seq |  | Single cell | 2016 | MCF-7 cells, mixed human MCF-7 and mouse 3T3 fibroblasts, primary CLL patient cells. | ~16000 | thousands | barcode | ^12^ |
| seqFISH |  | Single cell | 2016 | Mouse hippocampus | 100~250 | ~200 | in situ hybridization | ^13^ |
| tomo-seq |  | Single cell | 2016 | Zebrafish  embryo | ~12000 | / | 3D tissue reconstruction | ^14^ |
| LCM-seq |  | Single cell | 2016 | Mouse spinal  cord | ~10000 | 1 | laser capture microdissection | ^15^ |
| smHCR |  | Molecule | 2016 | Cultured cells,  Zebrafish  Embryos,  Mouse brain  cells | 5 | 6 | in situ hybridization | ^16^ |
| RNA SPOTs |  | Single cell | 2017 | NIH/3T3  fibroblast cells,  ES-E14 cells | ~10000 | / | in situ hybridization | ^17^ |
| Geo-seq |  | Spot | 2017 | Mouse early  embryo, brain,  pathological  liver, and sperm  tissues | ~8000 | 1 | combining image and single-cell RNA sequencing | ^18^ |
| NICHE-seq |  | Single cell | 2017 | Mouse lymph node, spleen | / | ~15000 | combining image and single-cell RNA sequencing | ^19^ |
| clampFISH |  | Single cell | 2018 | human metastatic melanoma cell line | / | / | in situ hybridization | ^20^ |
| osmFISH |  | Single cell | 2018 | Mouse brain | 33 | ~5000 | in situ hybridization | ^21^ |
| STARmap |  | Single cell | 2018 | Mouse primary  visual cortex | ~160-1000 | ~1000 | in situ sequencing | ^22^ |
| Visium |  | Spot | 2019 | Mouse and human tissues | ~5000 | ~5000 | barcode |  |
| HDST |  | Single cell | 2019 | Mouse  olfactory bulb | ~17000 | ~160000 | barcode | ^23^ |
| seqFISH+ |  | Single cell | 2019 | NIH/3T3  fibroblast cells,  mouse brain | ~10000 | ~3000 | in situ hybridization | ^24^ |
| GaST-seq |  | Spot | 2019 | A. thaliana leaf | hundreds | thousands | Combining image and single-cell RNA sequencing | ^25^ |
| APEX-seq |  | Subcellular | 2019 | Living human embryonic kidney (HEK) 293T cells | ~3250 | / | Direct proximity labeling | ^26^ |
| Stereo-seq |  | Single cell | 2020 | mouse embryonic tissues, brain, cortex, liver | ~800 | ~280000 | barcode | ^27^ |
| Split-FISH |  | Single cell | 2020 | Mouse hepatocytes, brain, liver, kidney, ovary | ~320 | ~26000 | in situ hybridization | ^28^ |
| HybISS |  | Single cell | 2020 | Mouse brain, human brain | ~120 | / | in situ sequencing | ^29^ |
| Zipseq |  | Single cell | 2020 | mouse lymph nodes, primary mouse CD4 and CD8 T cells | ~3500 | ~7000 | combining image and single-cell RNA sequencing | ^30^ |
| Slide-SeqV2 |  | Single cell | 2021 | Mouse brain | thousands | / | barcode | ^31^ |
| seq-scope |  | Subcellular | 2021 | Mouse liver, colon | ~2000 | / | barcode | ^32^ |
| sci-Space |  | Single cell | 2021 | Mouse embryos | ~1200 | ~120000 | barcode | ^33^ |
| XYZeq |  | Single cell | 2021 | HEK 293T and NIH 3T3 cell mixtures, mouse liver/tumor, spleen/tumor samples | thousands | ~8750 | barcode | ^34^ |
| EASI-FISH |  | Single cell | 2021 | Mouse brain | ~17500 | thousands | in situ hybridization | ^35^ |
| par-seqFISH |  | Single cell | 2021 | Pseudomonas  Aeruginosa cells | hundreds | ~50000 | in situ hybridization | ^36^ |
| BOLORAMIS |  | Spot | 2021 | HeLa, MCF7,  NIH-3T3 and HMC3 cells, human iPSCs | ~100 | / | in situ sequencing | ^37^ |
| BARseq2 |  | Single cell | 2021 | Mouse brain | dozens | ~3300 | in situ sequencing | ^38^ |
| ExSeq |  | Subcellular | 2021 | Mouse brain, human breast | thousands | ~2400 | in situ sequencing | ^39^ |
| STRP-seq |  | Spot | 2021 | Mouse brain, lizard brain | ~8000 | / | 3D tissue reconstruction | ^40^ |
| PIC |  | Subcellular | 2021 | Mouse embryo, mixed-species cultures of human- and mouse-derived cell lines | ~10000 | dozens to thousands | In situ reverse transcription | ^41^ |
| Light-Seq |  | Spot | 2022 | mouse retina, mixed cell cultures (mouse 3T3, human HEK) | ~24000 | several to thousands | barcode | ^42^ |
| Perturb-map |  | Single cell | 2022 | Mouse model of lung cancer, | dozens | >4700 | barcode | ^43^ |
| Space-TREX |  | Single cell | 2022 | Mouse brain | ~2300 | thousands | barcode | ^44^ |
| CBSST-seq |  | Spot | 2022 | Mouse brain | ~3200 | / | barcode | ^45^ |
| STRS |  | Single cell | 2022 | Mouse tibialis anterior muscles, mouse REOV infected hearts | thousands | ~4000 | barcode | ^46^ |
| Pixel-seq |  | Single cell | 2022 | Mouse brain | ~23000 | ~15000 | barcode | ^47^ |
| STOmics-GenX |  | Single cell | 2022 | Human hepatocellular carcinoma samples | ~17500 | thousands | barcode | ^48^ |
| slide-TCR-seq |  | Spot | 2022 | Mouse spleen, human lymph node and tonsil, renal cell carcinoma, melanoma samples | / | / | barcode | ^49^ |
| Matrix-seq |  | Spot | 2022 | Mouse brain | ~3500 | 4900 | barcode | ^50^ |
| EEL FISH |  | Single cell | 2022 | Mouse brain, human brain | ~440 | ~128000 | in situ hybridization | ^51^ |
| WTA |  | Spot | 2022 | Human kidney, mouse embryo | ~6000-11000 | tens to hundreds | in situ hybridization | ^52^ |
| SMI |  | Single cell | 2022 | Human lung tissue, non-small cell lung cancer tissue, breast cancer biopsy | ~1000 | ~2 millions | in situ hybridization | ^53^ |
| Funseq |  | Single cell | 2022 | MCF10A cells; MDA-MB-231 cells | >10000 | 1 to ${10}^{4}$ | combining image and single-cell RNA sequencing | ^54^ |
| immuno-LCM-RNAseq |  | Spot | 2022 | Mouse small intestine | >10000 | tens to ~2300 | combining image and single-cell RNA sequencing | ^55^ |
| PuTi-spots |  | Spot | 2022 | Mouse liver | ~7000 | hundreds | combining image and single-cell RNA sequencing | ^56^ |
| Image-seq |  | Single cell | 2022 | Mouse bone marrow | ~8000 | thousands | combining image and single-cell RNA sequencing | ^57^ |
| SmT |  | Spot | 2023 | host A. thaliana leaf sections | ~16000 | ~5000 | barcode | ^58^ |
| xDbit |  | Single cell | 2023 | Mouse cerebellum, liver, kidney, spleen, and heart | ~1000-5000 | ~1400 | barcode | ^59^ |
| Spatial VDJ |  | Spot | 2023 | Human tonsil, breast, lymphoid tissue | / | thousands | barcode | ^60^ |
| Ex-ST |  | Spot | 2023 | Mouse brain | hundreds | ~5000 | barcode | ^61^ |
| IISS |  | Single cell | 2023 | Mouse brain, human colon cancer, MCF-7 cell samples | dozens | / | in situ sequencing | ^62^ |
| RIBOmap |  | Single cell | 2023 | HeLa cells, mouse brain | ~5000 | ~60000 | in situ sequencing | ^63^ |
| SPRINTseq |  | Subcellular | 2023 | Mouse brain | ~100 | ~110000 | in situ sequencing | ^64^ |
| TEMPOmap |  | Subcellular | 2023 | Hela cells, hiPSC-CMs, primary human skin cells | hundreds to thousands | thousands | in situ sequencing | ^65^ |
| STARmap PLUS |  | Single cell | 2023 | Adult mouse brain and spinal cord | ~1000 | ~55000 | in situ sequencing | ^66^ |
| 10X Visium HD |  | Single cell | 2024 | Human CRC and normal adjacent mucosal tissues | tens of thousands | ~80000 | barcode | ^67^ |
| Decoder-seq |  | Single cell | 2024 | Mouse brain, human renal cell carcinoma | thousands | thousands | barcode | ^68^ |
| DEI | Proteome | Subcellular | 2017 | Mouse brain, retina sections, breast tumor carcinoma tissue, BSC1 cells | / | / | fluorescence imaging | ^69^ |
| t-CyCIF |  | Single cell | 2018 | Human tonsil, skin, melanoma tissues, mouse breast cancer | tens to hundreds | thousands | fluorescence imaging | ^70^ |
| 4i |  | Single cell | 2018 | HeLa cells | 40 | thousands | fluorescence imaging | ^71^ |
| Immuno-SABER |  | Single cell | 2019 | BSC1 cells, human tonsil, mouse retina, HeLa cells | 10 | hundreds of thousands | fluorescence imaging | ^72^ |
| MIBI-TOF |  | Subcellular | 2019 | Human breast cancer, spleen, tonsil, gastrointestinal tract, melanoma tissues, colorectal cancer | ~40 | thousands | mass spectrometry | ^73^ |
| CODEX |  | Single cell | 2019 | Ductal adenocarcinoma breast tissue | > 50 | thousands | fluorescence imaging |  |
| IBEX |  | Single cell | 2020 | Mouse lymph nodes, spleen, thymus, lung, liver, small intestine | ~70 | tens of thousands | fluorescence imaging | ^74,75^ |
| IMC |  | Single cell | 2021 | Human hepatocellular carcinoma, healthy liver, mouse liver | ~40 | / | mass spectrometry | ^76^ |
| DVP |  | Single cell | 2022 | Human fallopian tube epithelium, U2OS cells, salivary  gland acinic cell carcinoma, primary melanoma tissue | ~5000 | hundreds | mass spectrometry | ^77^ |
| MICS |  | Subcellular | 2022 | GBM, HGSOC, PDAC, mouse spleen, healthy human tissues (lung, liver, pancreas, ovary, skin, thyroid, testis, pituitary gland, brain, kidney heart) | hundreds | ~12000 | fluorescence imaging | ^78^ |
| scDVP |  | Single cell | 2023 | Mouse liver | several - ~2700 | / | mass spectrometry | ^79^ |
| Nano-DESI MSI | Genome | Single cell | 2023 | Mouse brain | hundreds to thousands | / | mass spectrometry | ^80^ |
| RESI |  | Molecule | 2023 | Chinese hamster ovary cells, U2OS cells | 1 | thousands | fluorescence imaging | ^81^ |
| SUM-PAINT |  | Molecule | 2024 | Mouse brain, rat primary hippocampal neurons | ~40 | / | barcode | ^82^ |
| DNA Microscopy |  | Single cell | 2019 | MDA-MB 231 cells, BT-549 cells | several to dozens | / | in situ hybridization | ^83^ |
| Slide-DNA-seq |  | Single cell | 2022 | Mouse cerebellum, liver metastases, lung tumor, primary human colorectal tumor | ~25000 | thousands | barcode | ^84^ |
| sciMAP-ATAC | ATAC | Single cell | 2021 | Mouse brain cortex, human brain cortex | dozens to hundreds | ~8000 | ATAC-seq | ^85^ |
| Epigenomic MERFISH | Epigenome/ATAC | Single cell | 2022 | Embryonic and adult mouse brains | ~150 | ~4200 | in situ hybridization | ^86^ |
| LCM-ATAC seq |  | Single cell | 2023 | Mouse brain, lung, spleen tissues, healthy human lung | dozens to hundreds | ~50-1000 | in situ ATAC-seq | ^87^ |
| SCA-seq |  | Molecule | 2024 | Mouse 4T1 cell line, human HEK293T cell line | ~70000 | / | methylation labeling and proximity ligation | ^88^ |
| Spatial-ATAC-seq | ATAC | Spot | 2022 | Mouse brain, human brain, tonsil | ~100000 | ~57000 | In situ ATAC-seq | ^89^ |
| Spatial ATAC |  | Spot | 2023 | Mouse embryo, breast cancer | Several thousands | Several thousands | In situ ATAC-seq | ^90^ |
| MALDI-IMS | Proteome & metabolism | Single cell | 2014 | Mouse brain, liver, retinal tissue | tens of thousands | / | mass spectrometry | ^91^ |
| MIBI |  | Single cell | 2017 | Human breast carcinoma cell line / tissue | >7 | / | mass spectrometry | ^92^ |
| DSP | Transcriptome & proteome | Spot | 2020 | Lymphoid, colorectal tumor and autoimmune  tissues | 44 proteins, 1412 genes | 1-5000 | in situ hybridization | ^93^ |
| DBiT-seq |  | Single cell | 2020 | Mouse embryos | ~4200 genes, ~20 proteins | / | barcode | ^94^ |
| CAD-HCR |  | Single cell | 2022 | HeLa cells, MCF-7 cells | 9 proteins | / | in situ hybridization | ^95^ |
| MOSAICA |  | Single cell | 2022 | HEK293T-X cells, human colorectal adenocarcinoma (SW480) cells, | 10 genes, several proteins | thousands | fluorescence imaging | ^96^ |
| SM-Omics |  | Single cell | 2022 | Mouse brain, spleen, colorectal cancer | ~5200 genes, 6 proteins | / | barcode | ^97^ |
| CosMx SMI |  | Single cell | 2022 | Human lung cancer, breast cancer, 51 cell lines | ~1000 RNAs, ~100 proteins | ~2 millions | in situ hybridization | ^53^ |
| Spatial-CITE-seq |  | Single cell | 2022 | Mouse spleen, colon, intestine, kidney, Human tonsil, skin | ~1200 genes, ~300 proteins | / | barcode | ^98^ |
| SPOTS |  | Spot | 2023 | Mouse spleen, liver | ~6000 genes, ~30 proteins | / | barcode | ^99^ |
| Stereo-CITE-Seq |  | Single cell | 2023 | Mouse spleen, thymus | dozens to thousands genes, hundreds proteins | / | barcode | ^100^ |
| Spatial ATAC-RNA-seq | Transcriptome & epigenome | Single cell | 2023 | Mouse brain, human brain | ~25000 genes | ~30000 | ATAC-seq | ^101^ |
| MISAR-seq |  | Spot | 2023 | Mouse brain | ~10000 genes | <75000 | barcode | ^102^ |
| Spatial-CITE-Seq | Epigenome & proteome | Single cell | 2023 | Mouse spleen, colon, intestine, kidney, Human tonsil, skin | ~1200 genes, ~300 proteins | / | barcode | ^103^ |
| Spatial-CUT & Tag | Transcriptome & epigenome & proteome | Single cell | 2022 | Mouse brain | ~20000 unique fragments | / | in situ sequencing | ^104^ |

**Supplementary Table 1. Summary of spatial multi-omics sequencing technologies.** Note: “/” indicates that no clear description is provided in the manuscript.

**References**

1 Wang, F. *et al.* RNAscope: A Novel in Situ RNA Analysis Platform for Formalin-Fixed, Paraffin-Embedded Tissues. *The Journal of Molecular Diagnostics* **14**, 22-29 (2012). <https://doi.org:https://doi.org/10.1016/j.jmoldx.2011.08.002>

2 Lovatt, D. *et al.* Transcriptome in vivo analysis (TIVA) of spatially defined single cells in live tissue. *Nature Methods* **11**, 190-196 (2014). <https://doi.org:10.1038/nmeth.2804>

3 Chen, K. H., Boettiger, A. N., Moffitt, J. R., Wang, S. & Zhuang, X. Spatially resolved, highly multiplexed RNA profiling in single cells. *Science* **348**, aaa6090 (2015). <https://doi.org:doi:10.1126/science.aaa6090>

4 Moffitt, J. R. *et al.* High-throughput single-cell gene-expression profiling with multiplexed error-robust fluorescence in situ hybridization. *Proceedings of the National Academy of Sciences* **113**, 11046-11051 (2016). <https://doi.org:doi:10.1073/pnas.1612826113>

5 Moffitt, J. R. *et al.* High-performance multiplexed fluorescence in situ hybridization in culture and tissue with matrix imprinting and clearing. *Proceedings of the National Academy of Sciences* **113**, 14456-14461 (2016). <https://doi.org:doi:10.1073/pnas.1617699113>

6 Moffitt, J. R. *et al.* Molecular, spatial, and functional single-cell profiling of the hypothalamic preoptic region. *Science* **362**, eaau5324 (2018). <https://doi.org:doi:10.1126/science.aau5324>

7 Xia, C., Fan, J., Emanuel, G., Hao, J. & Zhuang, X. Spatial transcriptome profiling by MERFISH reveals subcellular RNA compartmentalization and cell cycle-dependent gene expression. *Proceedings of the National Academy of Sciences* **116**, 19490-19499 (2019). <https://doi.org:doi:10.1073/pnas.1912459116>

8 Lee, J. H. *et al.* Highly Multiplexed Subcellular RNA Sequencing in Situ. *Science* **343**, 1360-1363 (2014). <https://doi.org:doi:10.1126/science.1250212>

9 Lee, J. H. *et al.* Fluorescent in situ sequencing (FISSEQ) of RNA for gene expression profiling in intact cells and tissues. *Nature Protocols* **10**, 442-458 (2015). <https://doi.org:10.1038/nprot.2014.191>

10 Macosko, Evan Z. *et al.* Highly Parallel Genome-wide Expression Profiling of Individual Cells Using Nanoliter Droplets. *Cell* **161**, 1202-1214 (2015). <https://doi.org:https://doi.org/10.1016/j.cell.2015.05.002>

11 Ståhl, P. L. *et al.* Visualization and analysis of gene expression in tissue sections by spatial transcriptomics. *Science* **353**, 78-82 (2016). <https://doi.org:doi:10.1126/science.aaf2403>

12 Vickovic, S. *et al.* Massive and parallel expression profiling using microarrayed single-cell sequencing. *Nature Communications* **7**, 13182 (2016). <https://doi.org:10.1038/ncomms13182>

13 Shah, S., Lubeck, E., Zhou, W. & Cai, L. In Situ Transcription Profiling of Single Cells Reveals Spatial Organization of Cells in the Mouse Hippocampus. *Neuron* **92**, 342-357 (2016). <https://doi.org:10.1016/j.neuron.2016.10.001>

14 Kruse, F., Junker, J. P., van Oudenaarden, A. & Bakkers, J. Tomo-seq: A method to obtain genome-wide expression data with spatial resolution. *Methods Cell Biol* **135**, 299-307 (2016). <https://doi.org:10.1016/bs.mcb.2016.01.006>

15 Nichterwitz, S. *et al.* Laser capture microscopy coupled with Smart-seq2 for precise spatial transcriptomic profiling. *Nature Communications* **7**, 12139 (2016). <https://doi.org:10.1038/ncomms12139>

16 Shah, S. *et al.* Single-molecule RNA detection at depth by hybridization chain reaction and tissue hydrogel embedding and clearing. *Development* **143**, 2862-2867 (2016). <https://doi.org:10.1242/dev.138560>

17 Eng, C.-H. L., Shah, S., Thomassie, J. & Cai, L. Profiling the transcriptome with RNA SPOTs. *Nature Methods* **14**, 1153-1155 (2017). <https://doi.org:10.1038/nmeth.4500>

18 Chen, J. *et al.* Spatial transcriptomic analysis of cryosectioned tissue samples with Geo-seq. *Nature Protocols* **12**, 566-580 (2017). <https://doi.org:10.1038/nprot.2017.003>

19 Medaglia, C. *et al.* Spatial reconstruction of immune niches by combining photoactivatable reporters and scRNA-seq. *Science* **358**, 1622-1626 (2017). <https://doi.org:10.1126/science.aao4277>

20 Rouhanifard, S. H. *et al.* ClampFISH detects individual nucleic acid molecules using click chemistry–based amplification. *Nature Biotechnology* **37**, 84-89 (2019). <https://doi.org:10.1038/nbt.4286>

21 Codeluppi, S. *et al.* Spatial organization of the somatosensory cortex revealed by osmFISH. *Nature Methods* **15**, 932-935 (2018). <https://doi.org:10.1038/s41592-018-0175-z>

22 Wang, X. *et al.* Three-dimensional intact-tissue sequencing of single-cell transcriptional states. *Science* **361**, eaat5691 (2018). <https://doi.org:doi:10.1126/science.aat5691>

23 Vickovic, S. *et al.* High-definition spatial transcriptomics for in situ tissue profiling. *Nat Methods* **16**, 987-990 (2019). <https://doi.org:10.1038/s41592-019-0548-y>

24 Eng, C.-H. L. *et al.* Transcriptome-scale super-resolved imaging in tissues by RNA seqFISH+. *Nature* **568**, 235-239 (2019). <https://doi.org:10.1038/s41586-019-1049-y>

25 Giolai, M. *et al.* Spatially resolved transcriptomics reveals plant host responses to pathogens. *Plant Methods* **15**, 114 (2019). <https://doi.org:10.1186/s13007-019-0498-5>

26 Fazal, F. M. *et al.* Atlas of Subcellular RNA Localization Revealed by APEX-Seq. *Cell* **178**, 473-490.e426 (2019). <https://doi.org:10.1016/j.cell.2019.05.027>

27 Chen, A. *et al.* Spatiotemporal transcriptomic atlas of mouse organogenesis using DNA nanoball-patterned arrays. *Cell* **185**, 1777-1792.e1721 (2022). <https://doi.org:10.1016/j.cell.2022.04.003>

28 Goh, J. J. L. *et al.* Highly specific multiplexed RNA imaging in tissues with split-FISH. *Nature Methods* **17**, 689-693 (2020). <https://doi.org:10.1038/s41592-020-0858-0>

29 Gyllborg, D. *et al.* Hybridization-based in situ sequencing (HybISS) for spatially resolved transcriptomics in human and mouse brain tissue. *Nucleic Acids Research* **48**, e112-e112 (2020). <https://doi.org:10.1093/nar/gkaa792>

30 Hu, K. H. *et al.* ZipSeq: barcoding for real-time mapping of single cell transcriptomes. *Nature Methods* **17**, 833-843 (2020). <https://doi.org:10.1038/s41592-020-0880-2>

31 Stickels, R. R. *et al.* Highly sensitive spatial transcriptomics at near-cellular resolution with Slide-seqV2. *Nat Biotechnol* **39**, 313-319 (2021). <https://doi.org:10.1038/s41587-020-0739-1>

32 Cho, C.-S. *et al.* Microscopic examination of spatial transcriptome using Seq-Scope. *Cell* **184**, 3559-3572.e3522 (2021). <https://doi.org:https://doi.org/10.1016/j.cell.2021.05.010>

33 Srivatsan, S. R. *et al.* Embryo-scale, single-cell spatial transcriptomics. *Science* **373**, 111-117 (2021). <https://doi.org:doi:10.1126/science.abb9536>

34 Lee, Y. *et al.* XYZeq: Spatially resolved single-cell RNA sequencing reveals expression heterogeneity in the tumor microenvironment. *Science Advances* **7**, eabg4755 (2021). <https://doi.org:doi:10.1126/sciadv.abg4755>

35 Wang, Y. *et al.* EASI-FISH for thick tissue defines lateral hypothalamus spatio-molecular organization. *Cell* **184**, 6361-6377.e6324 (2021). <https://doi.org:https://doi.org/10.1016/j.cell.2021.11.024>

36 Dar, D., Dar, N., Cai, L. & Newman, D. K. Spatial transcriptomics of planktonic and sessile bacterial populations at single-cell resolution. *Science* **373**, eabi4882 (2021). <https://doi.org:doi:10.1126/science.abi4882>

37 Liu, S. *et al.* Barcoded oligonucleotides ligated on RNA amplified for multiplexed and parallel in situ analyses. *Nucleic Acids Res* **49**, e58 (2021). <https://doi.org:10.1093/nar/gkab120>

38 Sun, Y.-C. *et al.* Integrating barcoded neuroanatomy with spatial transcriptional profiling enables identification of gene correlates of projections. *Nature Neuroscience* **24**, 873-885 (2021). <https://doi.org:10.1038/s41593-021-00842-4>

39 Alon, S. *et al.* Expansion sequencing: Spatially precise in situ transcriptomics in intact biological systems. *Science* **371**, eaax2656 (2021). <https://doi.org:doi:10.1126/science.aax2656>

40 Schede, H. H. *et al.* Spatial tissue profiling by imaging-free molecular tomography. *Nature Biotechnology* **39**, 968-977 (2021). <https://doi.org:10.1038/s41587-021-00879-7>

41 Honda, M. *et al.* High-depth spatial transcriptome analysis by photo-isolation chemistry. *Nature Communications* **12**, 4416 (2021). <https://doi.org:10.1038/s41467-021-24691-8>

42 Kishi, J. Y. *et al.* Light-Seq: light-directed in situ barcoding of biomolecules in fixed cells and tissues for spatially indexed sequencing. *Nature Methods* **19**, 1393-1402 (2022). <https://doi.org:10.1038/s41592-022-01604-1>

43 Dhainaut, M. *et al.* Spatial CRISPR genomics identifies regulators of the tumor microenvironment. *Cell* **185**, 1223-1239.e1220 (2022). <https://doi.org:https://doi.org/10.1016/j.cell.2022.02.015>

44 Ratz, M. *et al.* Clonal relations in the mouse brain revealed by single-cell and spatial transcriptomics. *Nature Neuroscience* **25**, 285-294 (2022). <https://doi.org:10.1038/s41593-022-01011-x>

45 Jin, Z. *et al.* Cross-amplified Barcodes on Slides for Spatial Transcriptomics Sequencing. *bioRxiv* (2022).

46 McKellar, D. W. *et al.* Spatial mapping of the total transcriptome by in situ polyadenylation. *Nature Biotechnology* **41**, 513 - 520 (2022).

47 Fu, X. *et al.* Polony gels enable amplifiable DNA stamping and spatial transcriptomics of chronic pain. *Cell* **185**, 4621-4633.e4617 (2022). <https://doi.org:https://doi.org/10.1016/j.cell.2022.10.021>

48 Currenti, J. *et al.* STOmics-GenX: CRISPR based approach to improve cell identity specific gene detection from spatially resolved transcriptomics. *bioRxiv*, 2022.2012.2008.519589 (2022). <https://doi.org:10.1101/2022.12.08.519589>

49 Liu, S. *et al.* Spatial maps of T&#xa0;cell receptors and transcriptomes reveal distinct immune niches and interactions in the adaptive immune response. *Immunity* **55**, 1940-1952.e1945 (2022). <https://doi.org:10.1016/j.immuni.2022.09.002>

50 Zhao, H., Tian, G. & Hu, A. Matrix-seq: An adjustable-resolution spatial transcriptomics via microfluidic matrix-based barcoding. *bioRxiv*, 2022.2008.2005.502952 (2022). <https://doi.org:10.1101/2022.08.05.502952>

51 Borm, L. E. *et al.* Scalable in situ single-cell profiling by electrophoretic capture of mRNA using EEL FISH. *Nature Biotechnology* **41**, 222-231 (2023). <https://doi.org:10.1038/s41587-022-01455-3>

52 Zimmerman, S. M. *et al.* Spatially resolved whole transcriptome profiling in human and mouse tissue using Digital Spatial Profiling. *Genome Res* **32**, 1892-1905 (2022). <https://doi.org:10.1101/gr.276206.121>

53 He, S. *et al.* High-plex imaging of RNA and proteins at subcellular resolution in fixed tissue by spatial molecular imaging. *Nature Biotechnology* **40**, 1794-1806 (2022). <https://doi.org:10.1038/s41587-022-01483-z>

54 Functional single-cell sequencing links dynamic phenotypes to their genotypes. *Nature Biomedical Engineering* **6**, 501-502 (2022). <https://doi.org:10.1038/s41551-022-00877-3>

55 Zhang, X. *et al.* Robust Acquisition of Spatial Transcriptional Programs in Tissues With Immunofluorescence-Guided Laser Capture Microdissection. *Front Cell Dev Biol* **10**, 853188 (2022). <https://doi.org:10.3389/fcell.2022.853188>

56 Matsunaga, H. *et al.* Reproducible and sensitive micro-tissue RNA sequencing from formalin-fixed paraffin-embedded tissues for spatial gene expression analysis. *Scientific Reports* **12**, 19511 (2022). <https://doi.org:10.1038/s41598-022-23651-6>

57 Haase, C. *et al.* Image-seq: spatially resolved single-cell sequencing guided by in situ and in vivo imaging. *Nature Methods* **19**, 1622-1633 (2022). <https://doi.org:10.1038/s41592-022-01673-2>

58 Saarenpää, S. *et al.* Spatial metatranscriptomics resolves host–bacteria–fungi interactomes. *Nature Biotechnology* **42**, 1384 - 1393 (2023).

59 Wirth, J. *et al.* Spatial transcriptomics using multiplexed deterministic barcoding in tissue. *Nature Communications* **14**, 1523 (2023). <https://doi.org:10.1038/s41467-023-37111-w>

60 Engblom, C. *et al.* Spatial transcriptomics of B cell and T cell receptors reveals lymphocyte clonal dynamics. *Science* **382**, eadf8486 (2023). <https://doi.org:doi:10.1126/science.adf8486>

61 Fan, Y. *et al.* Expansion spatial transcriptomics. *Nat Methods* **20**, 1179-1182 (2023). <https://doi.org:10.1038/s41592-023-01911-1>

62 Tang, X. *et al.* Improved in situ sequencing for high-resolution targeted spatial transcriptomic analysis in tissue sections. *J Genet Genomics* **50**, 652-660 (2023). <https://doi.org:10.1016/j.jgg.2023.02.004>

63 Zeng, H. *et al.* Spatially resolved single-cell translatomics at molecular resolution. *Science* **380**, eadd3067 (2023). <https://doi.org:doi:10.1126/science.add3067>

64 Chang, T. *et al.* Rapid and signal crowdedness-robust in situ sequencing through hybrid block coding. *Proc Natl Acad Sci U S A* **120**, e2309227120 (2023). <https://doi.org:10.1073/pnas.2309227120>

65 Ren, J. *et al.* Spatiotemporally resolved transcriptomics reveals the subcellular RNA kinetic landscape. *Nature Methods* **20**, 695-705 (2023). <https://doi.org:10.1038/s41592-023-01829-8>

66 Shi, H. *et al.* Spatial atlas of the mouse central nervous system at molecular resolution. *Nature* **622**, 552-561 (2023). <https://doi.org:10.1038/s41586-023-06569-5>

67 Oliveira, M. F. *et al.* Characterization of immune cell populations in the tumor microenvironment of colorectal cancer using high definition spatial profiling. *bioRxiv*, 2024.2006.2004.597233 (2024). <https://doi.org:10.1101/2024.06.04.597233>

68 Cao, J. *et al.* Decoder-seq enhances mRNA capture efficiency in spatial RNA sequencing. *Nature Biotechnology* (2024). <https://doi.org:10.1038/s41587-023-02086-y>

69 Wang, Y. *et al.* Rapid Sequential in Situ Multiplexing with DNA Exchange Imaging in Neuronal Cells and Tissues. *Nano Lett* **17**, 6131-6139 (2017). <https://doi.org:10.1021/acs.nanolett.7b02716>

70 Lin, J. R. *et al.* Highly multiplexed immunofluorescence imaging of human tissues and tumors using t-CyCIF and conventional optical microscopes. *Elife* **7** (2018). <https://doi.org:10.7554/eLife.31657>

71 Gut, G., Herrmann, M. D. & Pelkmans, L. Multiplexed protein maps link subcellular organization to cellular states. *Science* **361**, eaar7042 (2018). <https://doi.org:doi:10.1126/science.aar7042>

72 Saka, S. K. *et al.* Immuno-SABER enables highly multiplexed and amplified protein imaging in tissues. *Nature Biotechnology* **37**, 1080-1090 (2019). <https://doi.org:10.1038/s41587-019-0207-y>

73 Keren, L. *et al.* MIBI-TOF: A multiplexed imaging platform relates cellular phenotypes and tissue structure. *Sci Adv* **5**, eaax5851 (2019). <https://doi.org:10.1126/sciadv.aax5851>

74 Radtke, A. J. *et al.* IBEX: A versatile multiplex optical imaging approach for deep phenotyping and spatial analysis of cells in complex tissues. *Proc Natl Acad Sci U S A* **117**, 33455-33465 (2020). <https://doi.org:10.1073/pnas.2018488117>

75 Radtke, A. J. *et al.* IBEX: an iterative immunolabeling and chemical bleaching method for high-content imaging of diverse tissues. *Nature Protocols* **17**, 378-401 (2022). <https://doi.org:10.1038/s41596-021-00644-9>

76 Sheng, J. *et al.* Topological analysis of hepatocellular carcinoma tumour microenvironment based on imaging mass cytometry reveals cellular neighbourhood regulated reversely by macrophages with different ontogeny. *Gut* **71**, 1176-1191 (2022). <https://doi.org:10.1136/gutjnl-2021-324339>

77 Mund, A. *et al.* Deep Visual Proteomics defines single-cell identity and heterogeneity. *Nature Biotechnology* **40**, 1231-1240 (2022). <https://doi.org:10.1038/s41587-022-01302-5>

78 Kinkhabwala, A. *et al.* MACSima imaging cyclic staining (MICS) technology reveals combinatorial target pairs for CAR T cell treatment of solid tumors. *Scientific Reports* **12**, 1911 (2022). <https://doi.org:10.1038/s41598-022-05841-4>

79 Rosenberger, F. A. *et al.* Spatial single-cell mass spectrometry defines zonation of the hepatocyte proteome. *Nature Methods* **20**, 1530-1536 (2023). <https://doi.org:10.1038/s41592-023-02007-6>

80 Yang, M. *et al.* Nano-DESI Mass Spectrometry Imaging of Proteoforms in Biological Tissues with High Spatial Resolution. *Analytical Chemistry* **95**, 5214-5222 (2023). <https://doi.org:10.1021/acs.analchem.2c04795>

81 Reinhardt, S. C. M. *et al.* Ångström-resolution fluorescence microscopy. *Nature* **617**, 711-716 (2023). <https://doi.org:10.1038/s41586-023-05925-9>

82 Unterauer, E. M. *et al.* Spatial proteomics in neurons at single-protein resolution. *Cell* **187**, 1785-1800.e1716 (2024). <https://doi.org:https://doi.org/10.1016/j.cell.2024.02.045>

83 Weinstein, J. A., Regev, A. & Zhang, F. DNA Microscopy: Optics-free Spatio-genetic Imaging by a Stand-Alone Chemical Reaction. *Cell* **178**, 229-241.e216 (2019). <https://doi.org:https://doi.org/10.1016/j.cell.2019.05.019>

84 Zhao, T. *et al.* Spatial genomics enables multi-modal study of clonal heterogeneity in tissues. *Nature* **601**, 85-91 (2022). <https://doi.org:10.1038/s41586-021-04217-4>

85 Thornton, C. A. *et al.* Spatially mapped single-cell chromatin accessibility. *Nature Communications* **12**, 1274 (2021). <https://doi.org:10.1038/s41467-021-21515-7>

86 Lu, T., Ang, C. E. & Zhuang, X. Spatially resolved epigenomic profiling of single cells in complex tissues. *Cell* **185**, 4448-4464.e4417 (2022). <https://doi.org:https://doi.org/10.1016/j.cell.2022.09.035>

87 Carraro, C. *et al.* Chromatin accessibility profiling of targeted cell populations with laser capture microdissection coupled to ATAC-seq. *Cell Reports Methods* **3** (2023). <https://doi.org:10.1016/j.crmeth.2023.100598>

88 Xie, Y. *et al.* Spatial chromatin accessibility sequencing resolves high-order spatial interactions of epigenomic markers. *Elife* **12** (2024). <https://doi.org:10.7554/eLife.87868>

89 Deng, Y. *et al.* Spatial profiling of chromatin accessibility in mouse and human tissues. *Nature* **609**, 375-383 (2022). <https://doi.org:10.1038/s41586-022-05094-1>

90 Llorens-Bobadilla, E. *et al.* Solid-phase capture and profiling of open chromatin by spatial ATAC. *Nat Biotechnol* **41**, 1085-1088 (2023). <https://doi.org:10.1038/s41587-022-01603-9>

91 Gessel, M. M., Norris, J. L. & Caprioli, R. M. MALDI imaging mass spectrometry: spatial molecular analysis to enable a new age of discovery. *J Proteomics* **107**, 71-82 (2014). <https://doi.org:10.1016/j.jprot.2014.03.021>

92 Rost, S. *et al.* Multiplexed ion beam imaging analysis for quantitation of protein expresssion in cancer tissue sections. *Laboratory Investigation* **97**, 992-1003 (2017). <https://doi.org:10.1038/labinvest.2017.50>

93 Merritt, C. R. *et al.* Multiplex digital spatial profiling of proteins and RNA in fixed tissue. *Nature Biotechnology* **38**, 586-599 (2020). <https://doi.org:10.1038/s41587-020-0472-9>

94 Liu, Y. *et al.* High-Spatial-Resolution Multi-Omics Sequencing via Deterministic Barcoding in Tissue. *Cell* **183**, 1665-1681.e1618 (2020). <https://doi.org:10.1016/j.cell.2020.10.026>

95 Liu, X. *et al.* Computer-aided design of reversible hybridization chain reaction (CAD-HCR) enables multiplexed single-cell spatial proteomics imaging. *Science Advances* **8**, eabk0133 (2022). <https://doi.org:doi:10.1126/sciadv.abk0133>

96 Vu, T. *et al.* Spatial transcriptomics using combinatorial fluorescence spectral and lifetime encoding, imaging and analysis. *Nature Communications* **13**, 169 (2022). <https://doi.org:10.1038/s41467-021-27798-0>

97 Vickovic, S. *et al.* SM-Omics is an automated platform for high-throughput spatial multi-omics. *Nature Communications* **13**, 795 (2022). <https://doi.org:10.1038/s41467-022-28445-y>

98 Liu, Y. *et al.* Spatial-CITE-seq: spatially resolved high-plex protein and whole transcriptome co-mapping. *Res Sq* (2022). <https://doi.org:10.21203/rs.3.rs-1499315/v1>

99 Ben-Chetrit, N. *et al.* Integration of whole transcriptome spatial profiling with protein markers. *Nature Biotechnology* **41**, 788-793 (2023). <https://doi.org:10.1038/s41587-022-01536-3>

100 Liao, S. *et al.* Integrated Spatial Transcriptomic and Proteomic Analysis of Fresh Frozen Tissue Based on Stereo-seq. *bioRxiv*, 2023.2004.2028.538364 (2023). <https://doi.org:10.1101/2023.04.28.538364>

101 Zhang, D. *et al.* Spatial epigenome–transcriptome co-profiling of mammalian tissues. *Nature* **616**, 113-122 (2023). <https://doi.org:10.1038/s41586-023-05795-1>

102 Jiang, F. *et al.* Simultaneous profiling of spatial gene expression and chromatin accessibility during mouse brain development. *Nature Methods* **20**, 1048-1057 (2023). <https://doi.org:10.1038/s41592-023-01884-1>

103 Liu, Y. *et al.* High-plex protein and whole transcriptome co-mapping at cellular resolution with spatial CITE-seq. *Nature Biotechnology* **41**, 1405-1409 (2023). <https://doi.org:10.1038/s41587-023-01676-0>

104 Deng, Y. *et al.* Spatial-CUT&amp;Tag: Spatially resolved chromatin modification profiling at the cellular level. *Science* **375**, 681-686 (2022). <https://doi.org:doi:10.1126/science.abg7216>
