# Supplementary material for: Unravelling tumour spatiotemporal heterogeneity using spatial multimodal data
Source: Clin Transl Med. 2025 May 7;15(5):e70331. doi: 10.1002/ctm2.70331 (PMC12059211; doi:10.1002/ctm2.70331)
Supplement: Supplementary file 2 — Supporting Information [file CTM2-15-e70331-s001.docx]

| **Categories** | **Method** | **Technical principle** | **Advantages** | **Limitations** | **Applications** | **Link** |
| --- | --- | --- | --- | --- | --- | --- |
| **Horizontal integration** | PASTE^1^ | PASTE uses a fused Gromov-Wasserstein optimal transport model to align spatial transcriptomics data across tissue slices and integrate them into a stacked 3D alignment | Provide a tradeoff between generating 3D spatial information and increasing the read coverage in 2D; High spatial coherence and statistical power; Elimination of Manual Gene Selection; Cross-Technology Applicability | Lack of histological image integration; Constraint of computational efficiency and scalability; Challenging in cross-tissue data integration | Cell type identification; Differential gene expression analysis; Spatial gene expression pattern recognition; Cell-cell communication inference; Tumor heterogeneity analysis | https://github.com/raphael-group/paste |
|  | Stich3D^2^ | STitch3D integrates multiple 2D spatial transcriptomics tissue slices using a graph attention network and deep learning to reconstruct comprehensive 3D tissue architectures | Enhanced downstream analyses; Robust performance across diverse datasets; Supervised spatial domain detection | Lack of histological image integration; Dependency on scRNA-seq references; Constraint of computational efficiency; Challenging in cross-tissue data integration | 3D spatial domain identification; 3D cell-type deconvolution; Spatial trajectory inference; Gene denoising; Generation of virtual tissue slice; Identification of spatially variable genes | https://github.com/YangLabHKUST/STitch3D |
|  | GraphST^3^ | GraphST leverages graph neural networks and self-supervised contrastive learning to integrate gene expression profiles and spatial information | Superior spatial clustering performance; Batch effect removed; Compatibility with multiple spatial transcriptomics platforms | Lack of histological image integration; Dependency on scRNA-seq references; Constraint of computational efficiency; Challenging in cross-tissue data integration | Spatial clustering; multi-sample integration; ST and scRNA-seq integration | https://github.com/JinmiaoChenLab/GraphST |
|  | SPIRAL^4^ | SPIRAL integrates spatial transcriptomics data by combining graph-based domain adaptation for batch effect removal with cluster-aware optimal transport for spatial alignment | Batch-effect correction; Generalizability to unseen data; Superior performance across diverse datasets | Lack of histological image integration; May produce unevenly distributed alignments within clusters; Constraint of computational efficiency | Batch effect removal; Expression enhancement; Unseen sample prediction | https://github.com/guott15/SPIRAL |
|  | STAligner^5^ | STAligner employs a graph attention auto-encoder and triplet learning to integrate spatial transcriptomics datasets | Batch effect correction; Compatibility with multiple spatial transcriptomics platforms; Efficient alignment process | Does not account for local flexible distortions; | Spatially aware batch correction; Spatial domain identification; Differential gene expression analysis; Developmental dynamics analysis | https://github.com/zhoux85/STAligner |
|  | SPACEL^6^ | SPACEL employs a multi-layer perceptron for cell type deconvolution, a graph convolutional network with adversarial learning for spatial domain identification, and a differential evolution algorithm for 3D tissue reconstruction | Each SPACEL module outperforms state-of-the-art methods in their respective tasks; Robust batch effect minimization; more precise alignment and preserving structural integrity | Dependency on cell type deconvolution; limiting in nonlinear alignment; SPACEL requires retraining facing new slices | Cell type Deconvolution; Spatial domain identification; 3D alignment of  multi-slices | https://github.com/QuKunLab/SPACEL |
|  | SLAT^7^ | SLAT employs graph neural networks and adversarial discriminator to align heterogeneous spatial omics slices | Suitable for large-scale applications; Adaptive to complex deformations | Input embedding dependency; Requiring further development to address alignment across slices | Spatial domain identification; Cell type annotation; Batch effect correction | https://github.com/gao-lab/SLAT |
|  | PRECAST^8^ | PRECAST employs conditional autoregressive models to capture spatial dependencies effects and Gaussian mixture model to capture biological effects | Batch effect correction; Scalable to large datasets; Adapts to different spatial resolutions | Loss of local spatial detail in low-resolution data; Initial reliance on normalization and batch correction | Spatial domain identification; Differential expression analysis; Conditional SVA; trajectory inference | https://cran.r-project.org/package=PRECAST |
|  | STELLAR^9^ | STELLAR utilizes a graph convolutional neural network to learn spatial and molecular features from annotated spatially resolved single-cell datasets, allowing for the discovery of novel cell types in unannotated datasets. | Novel cell type discovery; Scalability for spatial cell atlases | Reliance on nuclear segmentation; Dependency on feature-rich datasets | Cell type annotation; Novel cell type discovery; Revealing higher-order tissue structures | https://github.com/snap-stanford/stellar |
|  | ProFAST^10^ | ProFAST employs conditional autoregressive models to capture spatial dependencies effects and a Poisson or Gaussian framework to capture bio effects | Preservation of Spatial Correlation; Fast Processing for large-scale dataset | Missing nonlinear biological effects; May not fully leverage available manual annotations | Spatial domain identification; DE analysis; cell-cell interaction analysis | https://github.com/feiyoung/ProFAST |
|  | CellCharter^11^ | CellCharter obtains cell embeddings using variational autoencoders, then aggregates cell features with neighboring cells, finally employs a Gaussian mixture model for spatial clustering | Adaptable across different spatial omics technologies; Suitable for large-scale applications; Showing holistic view of cell states | Dependence on data quality and modality; Limited exploration of cell spatial dynamics | Spatial clustering; Cluster cell-type enrichment; Cluster shape characterization; Spatial interaction analysis | https://github.com/CSOgroup/cellcharter |
|  | MENDER^12^ | MENDER captures cellular neighborhood frequencies across multiple spatial ranges and integrates them into a unified representation. This allows MENDER to align and compare spatial features across slices | Suitable for large-scale applications; Adaptable across different technologies and tissue systems; Analyzing multiple tissue slices in spatial domain identification | Dependence on Leiden clustering; Limited adaptive spatial radius; | Identifies spatial domains; Detects condition-specific spatial signatures; Interpreted features; scalable to large data | https://github.com/yuanzhiyuan/MENDER |
|  | CytoCommunity^13^ | CytoCommunity integrates multiple slices by constructing a graph for each spatial omics slice, using differentiable graph pooling to aggregate cell information and align tissue cellular neighborhoods across different slices | An ingenious model has more accurate and stable results; Scalable to heterogeneous categorical data | Users must first estimate cell-type composition; Lack of temporal dynamics | Condition-specific TCN discovery; Cell-cell communication inference; Spatial domain alignment across slices; Comparing the spatial organization of tissues across different conditions | https://github.com/tanlabcode/CytoCommunity |
|  | scPROTEIN^14^ | scPROTEIN builds spatially informative graphs that connect cells based on their spatial coordinates. These cell graphs are then combined into a unified representation, allowing scPROTEIN to integrate information across slices | Peptide-to-Protein aggregation; Wide applicability across MS-based and antibody-based proteomics datasets; Capable of handling noisy data | Cannot be applied to MS acquisition platforms; No ground truth for peptide uncertainty | Cell clustering; Batch-effect correction; Label transfer; Clinical analysis | https://github.com/TencentAILabHealthcare/scPROTEIN |
| **Vertical integration** | CellCharter^11^ | CellCharter obtains cell embeddings using variational autoencoders, then aggregates cell features with neighboring cells, finally employs a Gaussian mixture model for spatial clustering | Adaptable across different spatial omics technologies; Suitable for large-scale applications; Showing holistic view of cell states | Dependence on data quality and modality; Limited exploration of cell spatial dynamics | Spatial clustering; Cluster cell-type enrichment; Cluster shape characterization; Spatial interaction analysis | https://github.com/CSOgroup/cellcharter |
|  | MaxFuse^15^ | MaxFuse performs cross-modal integration by constructing fuzzy nearest-neighbor graphs for each modality to smooth linked features, and iteratively learning joint embedding using canonical correlation analysis | Designed for Weak Linkage; Lower Computational Cost; MaxFuse can initialize domain knowledge and results from other integration methods | Dependent on linked features | MaxFuse performs tri-modal integration with HUBMAP data | https://github.com/shuxiaoc/maxfuse |
|  | moscot^16^ | Moscot leverages optimal transport to map data across time and space. It computes transport costs between cells in different conditions, integrates multi-modal data using shared latent spaces | Suitable for large-scale applications; Frameworks to handle spatiotemporal data; Can accurately reconstructs differentiation trajectories | Not suitable for out-of-sample data points | moscot reconstructs atlas-scale developmental trajectories of the mouse embryogenesis atlas example | https://moscot-tools.org/ |
|  | SpatialGlue^17^ | A graph neural network model with a dual-attention mechanism by integration spatial locations and multi-omics data | Effective for handling high-dimensional spatial data | Adding new layers of omics data increases model complexity and runtime | SpatialGlue captures more anatomical details and scales well with data size | https://github.com/JinmiaoChenLab/SpatialGlue |
| **Diagonal integration** | SLAT^18^ | Graph adversarial learning algorithm for aligning heterogeneous slice data | Lightweight model, and enables aligning slices from different technologies | The weight of neighboring nodes is the same, limiting ability to align heterogeneous sample | On 3D reconstruction, SLAT is better equipped to account for non-rigid structural shifting and alteration among slices, enabling adaptive correction for potential deformation artifacts | <https://github.com/gao-lab/SLAT> |

**Supplementary Table 2. Summary of spatial multi-omics integration methods.**

**References**

1 Zeira, R., Land, M., Strzalkowski, A. & Raphael, B. J. Alignment and integration of spatial transcriptomics data. *Nature Methods* **19**, 567-575, doi:10.1038/s41592-022-01459-6 (2022).

2 Wang, G. *et al.* Construction of a 3D whole organism spatial atlas by joint modelling of multiple slices with deep neural networks. *Nat Mach Intell* **5**, 1200-1213 (2023).

3 Long, Y. *et al.* Spatially informed clustering, integration, and deconvolution of spatial transcriptomics with GraphST. *Nature Communications* **14**, doi:10.1038/s41467-023-36796-3 (2023).

4 Guo, T. *et al.* SPIRAL: integrating and aligning spatially resolved transcriptomics data across different experiments, conditions, and technologies. *Genome Biology* **24**, doi:10.1186/s13059-023-03078-6 (2023).

5 Zhou, X., Dong, K. & Zhang, S. Integrating spatial transcriptomics data across different conditions, technologies and developmental stages. *Nature Computational Science* **3**, 894-906 (2023).

6 Xu, H. *et al.* SPACEL: deep learning-based characterization of spatial transcriptome architectures. *Nature Communications* **14**, doi:10.1038/s41467-023-43220-3 (2023).

7 Xia, C.-R., Cao, Z.-J., Tu, X.-M. & Gao, G. Spatial-linked alignment tool (SLAT) for aligning heterogenous slices. *Nature Communications* **14**, doi:10.1038/s41467-023-43105-5 (2023).

8 Liu, W. *et al.* Probabilistic embedding, clustering, and alignment for integrating spatial transcriptomics data with PRECAST. *Nature Communications* **14**, doi:10.1038/s41467-023-35947-w (2023).

9 Brbić, M. *et al.* Annotation of spatially resolved single-cell data with STELLAR. *Nature Methods* **19**, 1411-1418, doi:10.1038/s41592-022-01651-8 (2022).

10 Liu, W. *et al.* ProFAST: a fast and scalable factor analysis for spatially aware dimension reduction of multi-section spatial transcriptomics data. *bioRxiv*, 2023.2007. 2011.548486 (2023).

11 Varrone, M., Tavernari, D., Santamaria-Martínez, A., Walsh, L. A. & Ciriello, G. CellCharter reveals spatial cell niches associated with tissue remodeling and cell plasticity. *Nature Genetics* **56**, 74-84, doi:10.1038/s41588-023-01588-4 (2023).

12 Yuan, Z. MENDER: fast and scalable tissue structure identification in spatial omics data. *Nature Communications* **15**, doi:10.1038/s41467-023-44367-9 (2024).

13 Hu, Y. *et al.* Unsupervised and supervised discovery of tissue cellular neighborhoods from cell phenotypes. *Nature Methods* **21**, 267-278, doi:10.1038/s41592-023-02124-2 (2024).

14 Li, W. *et al.* scPROTEIN: a versatile deep graph contrastive learning framework for single-cell proteomics embedding. *Nature Methods* **21**, 623-634, doi:10.1038/s41592-024-02214-9 (2024).

15 Chen, S. *et al.* Integration of spatial and single-cell data across modalities with weakly linked features. *Nature Biotechnology* **42**, 1096-1106, doi:10.1038/s41587-023-01935-0 (2023).

16 Klein, D. *et al.* Mapping cells through time and space with moscot. *bioRxiv*, 2023.2005. 2011.540374 (2023).

17 Long, Y. *et al.* Deciphering spatial domains from spatial multi-omics with SpatialGlue. *Nat Methods*, 1-10 (2024).

18 Xia, C. R., Cao, Z. J., Tu, X. M. & Gao, G. Spatial-linked alignment tool (SLAT) for aligning heterogenous slices. *Nat Commun* **14**, 7236, doi:10.1038/s41467-023-43105-5 (2023).
