# Supplementary material for: Unravelling tumour spatiotemporal heterogeneity using spatial multimodal data
Source: Clin Transl Med. 2025 May 7;15(5):e70331. doi: 10.1002/ctm2.70331 (PMC12059211; doi:10.1002/ctm2.70331)
Supplement: Supplementary file 3 — Supporting Information [file CTM2-15-e70331-s002.docx]

| **Categories** | **Method** | **Technical principle** | **Advantages** | **Limitations** | **Applications** | **Link** |
| --- | --- | --- | --- | --- | --- | --- |
| **Spatial clustering** | Louvain^1^ | Graph-based non-spatial clustering, optimizing modularity | Fast execution, suitable for large-scale data | Does not consider spatial information, and lacks spatial continuity | Basic clustering for single-cell RNA-seq data, non-spatial baseline | https://scanpy.readthedocs.io/en/stable/generated/scanpy.tl.louvain.html |
|  | Leiden^1,2^ | Similar to Louvain, further improves the robustness of community detection | More robust clustering results compared to Louvain | Does not consider spatial information, and is unable to capture spatial patterns | Similar to Louvain, suitable for large-scale non-spatial data | https://scanpy.readthedocs.io/en/stable/generated/scanpy.tl.leiden.html |
|  | SpaGCN^3^ | Graph Convolutional Network integrates gene expression, spatial location, and histology data to learn cell/spot features | Constructing cell/spot graph by combining spatial locations and histological color features | The grayscale features in histological images are not fully utilized | Spatial domain identification and subsequent domain guided differential expression analysis | https://github.com/jianhuupenn/SpaGCN |
|  | BayesSpace^4^ | Bayesian statistical model encouraging neighboring points to belong to the same cluster | Strong at handling multi-scale spatial information | Requires high computational resources and extended runtime | Spatial domain identification | https://github.com/edward130603/BayesSpace |
|  | stLearn ^5^ | Graph-based framework integrating morphology, gene expression and spatial locations and pseudo-time-space to model and uncover relations between spatial domain | Inferring the trajectories that recapitulate changes in biological processes connecting neighboring cells across tissues | Can not accurately detect the boundary | Spatial domain identification and infer relations between cell-states across tissues undergoing dynamic change | https://stlearn.readthedocs.io/ |
|  | SEDR^6^ | Deep autoencoder combined with a mask self-supervised learning to construct cell representation | Automatically learns low-dimensional embeddings | High computational complexity, not suitable for large datasets | Spatial clustering, batch integration, trajectory inference, gene expression imputation, and denoising | https://github.com/JinmiaoChenLab/SEDR/ |
|  | CCST^7^ | Encodes gene expression and spatial location using Deep Graph Infomax, followed by PCA for clustering. | CCST enables learning cell features that capture node-level and graph-level structures | Unable to accurately detect the boundary | Spatial clustering, identify cell groups of all four cell cycle phases and spatial proximity between adjacent phases | https://github.com/xiaoyeye/CCST |
|  | STAGATE^8^ | A graph attention network combines gene expression and spatial location for learning low-dimensional embeddings | High efficiency and scalability | Time consuming for large-scale datasets | Spatial clustering, and 3D reconstruction | https://stagate.readthedocs.io/en/latest/index.html |
|  | stMVC^9^ | Multi-view clustering method that integrates gene expression, spatial locations, histological images, and manual annotations | Effectively integrate histological features and tumor contexts for dissecting intra-tumoral heterogeneity | Challenging to dissect internal heterogeneity within one tumor region | identifies spatial domains and transited cell-states | https://github.com/cmzuo11/stMVC |
|  | SpaceFlow^10^ | Learn cell embeddings by combining gene expression and spatial information through spatially regularized graph neural networks | Unravel spatiotemporal patterns of cells through pseudo-Spatiotemporal Map on cell embeddings | Can not accurately detect the boundary | Identifies spatial domains, and infers spatially contiguous trajectories | https://github.com/hongleir/SpaceFlow |
|  | conST^11^ | Contrastive learning framework integrating gene expression, spatial, and morphological information for embedding | Handles multi-modal data well, high-quality embeddings | High computational complexity, long training time | Ideal for complex multi-modal data scenarios, especially in noisy environments | https://github.com/ys-zong/conST |
|  | DR-SC^12^ | Combines dimension reduction and spatial clustering in a unified framework using a hidden Markov random field model | Simultaneously performs dimension reduction and clustering | Computationally intensive when dealing with very large datasets | Spatial domain identification, trajectory inference, and detection of gene expression with spatial variations | https://github.com/feiyoung/DR-SC.Analysis |
|  | stKeep^13^ | A heterogeneous graph learning approach for analyzing SRT data to dissect tumor ecosystems by identifying cell-modules, gene-modules, and CCC, based on multimodal data | Learning cell/spot features by combining local relations between cell, gene, and histological regions, as well as global semantic relations between cells | Identifying the histological regions from histological images may be time-consuming | stKeep detects more TME-related cell-state | https://github.com/cmzuo11/stKeep/ |
|  | BASS^14^ | Bayesian hierarchical model-based multi-scale and multi-sample analysis | Excellent performance in multi-scale scenarios, robust | Computationally intensive when dealing with very large datasets | Applied to multi-scale datasets, particularly those with significant spatial differences | https://github.com/zhengli09/BASS |
|  | GraphST^15^ | Self-supervised contrastive learning with graph modeling gene expression and spatial information | Effectively utilizes gene expression and spatial information, ensuring strong spatial consistency | High computational requirements for large-scale dataset | Spatial clustering, and jointly analyze multi-slices in vertical and horizontal integration while correcting batch-effects | https://github.com/JinmiaoChenLab/GraphST |
| **Identification of SVG** | trendsceek^16^ | Uses a permutation process to estimate spatial dependency | Simple and intuitive, suitable for standard analysis | High computational requirements for large-scale dataset | Identification of spatially variable genes based on permutations | https://github.com/edsgard/trendsceek |
|  | SpatialDE^17^ | Leverages Gaussian process regression to assess spatial variance | Captures complex spatial patterns and nonlinear structures | Computationally intensive, requires multiple model parameters | SpatialDE is not limited to sequencing technologies and can be applied to any expression data with spatial and/or temporal annotation | https://github.com/Teichlab/SpatialDE |
|  | SPARK-X ^18^ | SPARK-X is a scalable non-parametric test for spatial expression patterns analysis | SPARK-X relies on non-parametric covariance test for detecting spatial expression patterns | SPARK-X currently has limited support for the use of other covariance functions and transformations in a computationally efficient manner | SPARK-X performs robustly on large-scale, sparse spatial transcriptomics data | https://github.com/xzhoulab/SPARK |
|  | SOMDE^19^ | Utilizes self-organizing maps to identify spatially variable genes | Handles high-dimensional data well, suitable for various datasets | Complex model with long training times | SOMDE enhances the spatial patterns of SVG expressions, which helps for better visualization | https://github.com/XuegongLab/somde |
|  | Hotspot^20^ | Uses graph models to analyze spatial patterns | Captures complex spatial structures and gene interactions | Relies on graph construction, leading to high computational costs | Instead of constructing a similarity metric, it identifies genes that align with a given metric and groups them into modules | http://www.github.com/Yoseflab/Hotspot |
|  | SpaGCN^21^ | SpaGCN integrates gene expression, spatial location, and histology to model spatial dependency of gene expression for the identification of spatial domains and domain enriched SVGs | SpaGCN is computationally fast and memory efficient | SpaGCN is lack of separation of spatial variation and cell type variation in gene expression patterns for the detected SVGs | The SpaGCN-detected SVGs are transferrable and can be utilized for downstream analyses in independent tissue sections | https://github.com/jianhuupenn/SpaGCN |
|  | STAMarker^22^ | Uses saliency maps for spatially variable gene detection | STAMarker considers all genes simultaneously, allowing it to leverage the complementary information across genes | Heavily reliant on prior spatial clustering performance | STAMarker robustly identifies SVGs even when the data is down sampled to a very sparse level | http://github.com/zhanglabtools/STAMarker |
|  | BSP^23^ | leverages a big-small patch algorithm to identify SVGs at varying levels | Handles various resolution spatial data | High model complexity, sensitive to data scale and resolution | BSP processes 3D spatial data | https://github.com/juexinwang/BSP/ |
|  | PROST ^24^ | Uses a PI indicator to evaluate spatial expression variation | For high spatial resolution ST data, PROST outperforms existing methods in identifying subtle tissue structures | Overlooks tissue context from histological images, relying primarily on spatial gene expression for domain segmentation | Enhances spatial domain detection | https://github.com/Tang-Lab-super/PROST |
| **Cell type deconvolution** | Stereoscope^25^ | A negative binomial model estimates gene expression profiles in single-cell data and spot cell type abundances in STR data using MLE | The results are highly interpretable; suitable for working with complex tissues populated by several similar cell types | Requires paired single-cell data; limiting proportion estimates to the cell types represented in that data | Cell type co-localization | https://github.com/almaan/stereoscope |
|  | DSTG^26^ | Apply a GCN to learn the link-graph constructed by single-cell data and spatial data in low-dimensional space and output cell type composition vectors for each node | DSTG simultaneously utilizes highly variable genes and neighbor graphical structures; DSTG performs well as sequencing depth increases | DSTG functions as a black-box AI model, and the method used to construct the link-graph affect outcomes | Cell-type decomposition | https://github.com/Su-informatics-lab/DSTG |
|  | SPOTlight^27^ | NMF-based model decomposes single-cell data to infer spot topics and cell type compositions in spatial transcriptomics using NNLS | Perform good with shallowly sequenced references; Have broad and flexible application spectrum in different bio logical scenarios and technology versions | The NMF algorithm used in SPOTlight is stochastic | Spatial domain identification; Cell type enrichment in tumoral and non- tumoral regions | https://github.com/MarcElosua/SPOTlight_deconvolution_analysis |
|  | SpatialDWLS^28^ | Enrichment analysis of cell-type-specific marker genes filters unlikely cell types for each spot, followed by weighted least squares to infer cell type composition | spatialDWLS enhancing specificity by removing irrelevant cell-types; Can be easily accessed in Giotto | Sensitivity to marker gene selection; | Assortative analysis to evaluate the degree of spatial coherence | https://github.com/RubD/Giotto |
|  | Tangram^29^ | Utilizes neural networks to learn mapping probabilities from single-cell data to ST spots and estimate cell density for each spot | Tangram can align snRNA-seq data onto different spatial omics data and have different aspects of benefits on genome-wide expansion, enhances lower-resolution, Error correction and Multimodal integration | Tangram required knowledge on (segmented) cell numbers | Expands high-resolution ST to genome scale; Accurate correction of transcripts; Detect spatial localization of chromatin-accessibility patterns and cell-type patterns | https://github.com/broadinstitute/Tangram |
|  | RCTD^30^ | A Poisson model estimates STR gene expression, using spot cell type proportions as parameters, with MLE providing deconvolution results. | The platform effect normalization procedure is robust and adaptable to various target platform | RCTD relies on an assumption that platform effects are shared among cell types; Cell types missing from the reference | Discovers spatial localization of cellular subtypes; Detection of spatially variable genes within cell  type | https://github.com/dmcable/RCTD |
|  | Cell2location^31^ | A negative binomial model infers reference gene expression, while variational inference estimates spot cell type abundances in STR data | Cell2location is highly sensitive, fast, robust, and versatile. Integrated with the scvi-tools framework, it supports diverse applications | Facing the challenge of adapting to higher-resolution ST technologies and addressing different noise characteristics | Cell-type enrichment in different regions; Constructing cell-cell interaction networks and cell-type spatial co-localization networks | https://github.com/BayraktarLab/cell2location/ |
|  | DestVI^32^ | A negative binomial model and variational autoencoding estimate gene and continuous subtype abundances, with penalized likelihood inferring spot cell type and subtype proportions | DestVI identifies cell-type-specific differential expression in spatial data, estimates gene expression for each cell type at each spot, and enforces smoothness in cell-type abundance across spatial coordinates | May suffer from the missing cell types in the reference scRNA-seq data | Detects cell-type-specific sub-states, learns transcriptomic modules for each cell type, and identifies key genes with cell-type-specific changes across conditions | https://github.com/romain-lopez/DestVI-reproducibility |
|  | STdecon^33^ | A latent Dirichlet model infers spot cell types and gene expression, using variational EM without requiring single-cell reference data | Adaptable to high-resolution multi-cellular pixel resolution ST data | STdecon may not be optimal for sub-cellular resolution ST data, and its accuracy may decrease with fewer than 10 pixels. Additionally, the LDA model relies on underlying assumptions | Spatial cell-type decomposition and get cell-type specific gene expression | https://github.com/JEFworks-Lab/STdeconvolve |
|  | CellPie^34^ | Non-negative matrix factorization jointly decomposes ST and spatial graph features, optimizing the shared spot-topic matrix via Frobenius norm minimization | CellPie is a fast, reference-free topic modelling method which integrates spatial gene expression counts data and histological imaging features | CellPie does not explicitly model the spatial nature of the data | Only spatial cell-type decomposition | https://github.com/ManchesterBioinference/CellPie. |
|  | CellsFromSpace^35^ | Independent component analysis extracts spot topics from spatial transcriptomics data without requiring a single-cell reference | CellsFromSpace supports for all commercially available ST technologies,  without need of high-quality reference datasets; Its Shiny UI is easy to use; Easily allows for the integrated analysis of multiple samples | Time-consuming manual curation of annotation of ICs; Unresolved 2D signal analysis; No cell type proportion inference | Spatial domain identification; marker genes definition; Pseudo-time analysis | https://github.com/gustaveroussy/CFS. |
|  | POLARIS^36^ | A negative binomial model predicts spot cell type composition, optimized by MLE and MAP; Image data can train a network for predicting new spot cell composition. | Integration of layer structure and histological images; Can do deconvolution using only image information; | Integration of histological images hindered by image quality; Layer relying on pathologist annotations | Identify layer specific DE genes; Zero-shot learning of spot decomposition based on new image | https://github.com/JiawenChenn/POLARIS |
|  | Redeconve^37^ | Minimizes the loss between spatial omics matrices and those constructed from single-cell data using quadratic programming | Redeconve outperforms in resolution, accuracy, sparsity, robustness, and computational speed; Enables deconvolution at single-cell resolution | High computational cost | Spatial domain identification; Differential gene expression analysis; Co-localization Network | https://github.com/ZxZhou4150/Redeconve |
|  | RETROFIT^38^ | Spatial gene expression is modeled with L components (NMF)， each component is annotated with cell types based on correlations with prior cell-type gene expression | Reference-free design； Accurate cell-type-specific gene expression estimates；Allows for flexible updates to cell-type annotations without re-running Broad applicability in disease research | Lack of spatial awareness; RETROFIT simplifies modeling by omitting spatial correlations | Infers cell-type-specific marker gene; Cell-type mapping; | https://bioconductor.org/packages/release/bioc/html/retrofit.html |
|  | scProjection^39^ | Trains VAEs for each reference cell type, maps ST spot expressions, and uses linear regression to estimate cell type proportions. | Enhances gene expression resolution; Distinguishes intra-cell type variation; accommodates rare cell types | Limited applicability without suitable atlases and requires careful experimental design | Detection of spatial gene expression patterns; Gene expression imputation; Separation of RNA contributions; Cell-cell and gene-gene network analysis | https://github.com/quon-titative-biology/scProjection |
|  | SPADE^40^ | Identifies domains with spaGCN, estimates the domain cell types via Lasso, and computes spot compositions using augmented Lagrangian minimization | Spatial domain consideration; Accurate cell type identification; Insight into developmental processes | Constrained by using a single scRNA-seq dataset as the reference | Cell type co-localization analysis; Temporal dynamics analysis; Marker gene expression patterns | https://github.com/anlingUA/SPADE |
|  | SpatialCTD^41^ | Builds graphs from spatial distance and gene expression data, and uses a graph encoder to predict unlabeled spot cell types | A Graph Neural Network-based approach that does not rely on single-cell RNA-seq data and can adapt to different spot sizes | Lack of single-cell resolution ST data integration | Spatial analysis of resolution enhanced gene expression; Tumor-immune interactions analysis | https://omicsml.github.io/SpatialCTD/ |
|  | SpatialPrompt^42^ | Estimates neighboring spot influence with non-negative ridge regression, then uses KNN to predict cell type composition from simulated spatial data | High speed; Robust performance across references; Integrates multiple scRNA-seq references effectively | Depends on reference quality, requires cell-type annotation, and necessitates careful adjustment of the number of neighbors | Spatial domain identification; Integration with scRNA-seq data; Microenvironment analysis | https://github.com/swainasish/SpatialPrompt. |
|  | STdGCN^43^ | Builds a link-graph between pseudo-ST and real ST data using MNN, then applies a graph convolutional network to predict cell type composition | Integrates spatial structure and is adaptable to different ST platforms | Exhibits lower predictive accuracy for cell types with lower proportions and depends on the pseudo-spot generation method | Cell type co-localization analysis; Spatial domain identification; Tumor microenvironment dissection; Developmental tissue analysis | https://github.com/luoyuanlab/stdgcn. |
|  | STIE^44^ | An EM algorithm optimizes the joint likelihood of gene expression and nuclear morphology, incorporating penalties for discrepancies in proportion estimates | Single-cell resolution; improved cell type deconvolution through nuclear morphology integration; reduced colocalization bias | STIE relies on accurate nucleus segmentation and requires further validation across diverse domains | Spatial domain identification; Spatial cell-cell interaction analysis; Cell-type colocalization | https://github.com/zhushijia/STIE. |
| **Inference of CCC** | Giotto^45^ | Methods based on ligand-receptor database and rank testing | Versatile data support and integration | Limited to known ligand-receptor pairs and assumes linear models | Predicts top-ranked spatial ligand-receptor pairs; identifies differentially expressed genes mediated by CCC | http://www.spatialgiotto.com |
|  | stLearn ^5^ |  | Multiview integration; Detection of Interaction Hotspots | Limited to known ligand-receptor pairs; data quality dependency | Identify high-activity cell communication regions | https://github.com/petersaj/histology) |
|  | SpaTalk^46^ |  | Downstream TFs were considered; Inferring CCC at single-cell resolution | Limited to known ligand-receptor pairs; relying on matched scRNA-seq and ST data | Decomposition; Identifies active downstream TF | https://github.com/ZJUFanLab/SpaTalk |
|  | CellChat V2^47^ |  | Incorporating the core interaction between ligands and receptors with multi-subunit  structure along with modulation by cofactors | Limited to known ligand-receptor pairs, with cross-condition analysis primarily restricted to pairwise comparisons | Infers and analyzes intercellular communication network | https://github.com/jinworks/CellChat). |
|  | SpatialDM^48^ |  | Both global and local CCC were considered; Scalable to big dataset | Limited to known ligand-receptor pairs; The  detection of differential communicating LR pairs between conditions  is challenging | Selection of interaction ligand-receptor pairs; Interaction pattern clustering; Pathway enrichment; Differential analysis | https://github.com/StatBiomed/SpatialDM |
|  | GCNG^49^ | Methods utilizing graph neural networks | Can predict novel spatial interaction gene pairs | More dependent on graph structure and lacks focus on specific cell types | Infers the direction of extracellular ligand-receptor interactions; Functional gene assignment | https://github.com/xiaoyeye/GCNG |
|  | HoloNet^50^ |  | Focused on functional communication events and models CCC from a multi-ligand-receptor perspective | Requiring prior knowledge embedding into the model | Provides CCC hotspots and cell-type-level CCC networks; Predicts specific gene expressions; Identifies the core mediators and core  senders | https://github.com/lhc17/HoloNet. |
|  | DeepLink^51^ |  | Highly efficient in learning from incomplete data and requires no prior knowledge | DeepLinc does not provide information on the direction or strength of cell interactions | Inferring the signature genes  contributing to the cell interaction landscapes; Re-clustering based  on the spatially coded cell heterogeneity | https://github.com/xryanglab/DeepLinc |
|  | NCEM^52^ |  | Considers the effects of niche composition on gene expression and can be applied to complex datasets, including 3D data | Requires accurate capture of niche heterogeneity | Deconvolution of subclusters; Type coupling analysis, and Sender-receiver effect analysis | https://github.com/theislab/ncem |
|  | Stkeep^53^ | Methods based on prior gene-gene network | Integrates prior CCC and TF-TG knowledge with cell state and is effective in dissecting the tumor microenvironment | Depends on the prior co-related gene network and does not account for the directional relationships between genes | Identifies key transcription factors, ligands, and receptors involved in disease progression | https://github.com/cmzuo11/stKeep |
|  | StMLnet^54^ |  | Models the L-R-TF-TG multilayer network and is effective on data with varying spatial resolutions and gene coverage | Relies on several simplified assumptions and depends on prior TF-target information | Inference of multilayer 20  signaling network; Inference of L-R targets | https://github.com/SunXQlab/stMLnet). |
|  | MISTy^55^ | Methods based on traditional statistical approaches | Focuses on multiplexed contexts to dissect different effects and utilizes an explainable machine learning framework | Depends on precise view selection, and the extracted CCCs cannot be considered directly | Extracts the contribution of different views to the expression of markers and estimates the marker interactions from each view | https://saezlab.github.io/mistyR/ |
|  | SVCA^56^ |  | Decomposes individual mRNA or protein into spatial and non-spatial components | Faces computational cost challenges, requires suitable processing of raw data, and the model is univariate | Identifies genes significantly affecting cell interaction effects; Enrichment analysis | https://github.com/damienArnol/svca |
|  | IGAN^57^ |  | The analysis extends to all genes, rather than just LRs, and measures CCCs between single-cell pairs | Relies on cell type annotations; incurs High computational costs with large dataset | Measures CCC activity at cell/spot resolution, discovers CCC spatial heterogeneity, and detects CCC patterns | https://github.com/Zhu-JC/IGAN |
|  | COMMOT^58^ | Methods Utilizing OT | Uses a collective OT method to combine the competition between different LRs and spatial distances | The accuracy depends on the selection of the OT cost function | Identifies differential CCC direction; Groups CCC networks; Detects DEGs related to CCC | https://github.com/zcang/COMMOT |
| **Pseudo-time-space analysis** | SIRV^59^ | SIRV integrates scRNA-seq and spatial transcriptomics data using domain adaptation and KNN regression to predict RNA velocity and spatial cell differentiation trajectories | Suitable for high-resolution imaging-based spatial data integration; Facilitates interpretation of unannotated spatial datasets | SIRV is only evaluated on a single dataset, limiting its generalizability, and the characterization of differentiation directions remains uncertain | Spatial cell annotation; Cellular differentiation direction; Spatial pattern identification | https://github.com/tabdelaal/SRV |
|  | stMVC^60^ | stMVC uses multi-view graph collaborative learning to identify spatial domain, and then infers the pseudo-temporal based on PAGA graph | Multi-layer integration, efficient feature extraction, adaptability to various datasets, and improved noise handling | Tumor region segmentation is manually annotated; Need integration with generalizable deep learning segmentation tools | Spatial domain detection; Cell-State identification; Denoising gene expression | https://github.com/cmzuo11/stMVC |
|  | STT^61^ | The STT model uses a transition tensor and parameter estimation to compute multistable attractors and cell transitions | Uncovering attractors underlying both gene expression and splicing dynamics; Robust to initial state; A transition tensor used instead of direct RNA velocity; Iterative self-consistency | Higher-order gene interactions are not considered; Multimodal data is not incorporated; Automatic detection of root and target states is challenging; Prior knowledge of cell differentiation potencies is required for better state detection | Identifying multiple stable cell states; Reconstructing the transition paths; Identifying genes and biological pathways that are most relevant to the multistable expression patterns | https://github.com/cliffzhou92/STT/tree/release |
|  | stLearn ^5^ | Pseudotime is first calculated via DPT, then combined with spatial distance to form PSTD. The directed minimum spanning tree is used for optimized pseudotime prediction | Analyzes relationships within sub-clusters at a local level and between clusters at a global level | High computational complexity, requires a user-defined root, and depends on preprocessing methods | Identifying pseudo-time related transition genes | https://github.com/petersaj/histology) |
|  | spaTrack^62^ | Optimal transport paths between cells are computed to infer pseudotime, considering spatial distance and gene expression differences as transport costs | Integrates spatial distance into cell transition costs for more accurate trajectory reconstruction, enabling precise single-cell trajectories | Requires substantial computing power and memory, with memory demands increasing exponentially as population size grows | Infers gene regulatory network; Captures developmental driven factors; Identifies tumor heterogeneity and metastasis tracking | https://github.com/yzf072/spaTrack |
|  | SpaceFlow^10^ | Learns cell embeddings by combining gene expression and spatial information through spatially regularized graph neural networks | Unravels spatiotemporal patterns of cells through pseudo-Spatiotemporal Map on cell embeddings | Can not accurately detect the boundary | Identifies spatial domains, and infers spatially contiguous trajectories | https://github.com/hongleir/SpaceFlow |
|  | Paella^63^ | Pseudotime values for each cell are rank-tested to construct a spatial directed graph, followed by an iterative algorithm to build spatial pseudo-trajectories | Decomposing complex cell trajectories into multiple spatial sub-trajectories; Can handle complex spatial structures | Depends on the accuracy of initial pseudo-time, has high computational complexity, and is sensitive to spatial node distribution | Identifies genes with differential temporal patterns, maps cell type progression in complex spatial patterns, and performs gene ontology enrichment | https://github.com/Winnie09/Paella |
|  | CalicoST^64^ | Infers allele-specific copy numbers and reconstructs a phylogeography relating cancer clones in time and space | Inferring spatial tumor evolution, progression, and metastasis from SRT data | Requires sufficient coverage of heterogeneous SNPs; constrained by sequencing depth; dependent on having enough LOH events | Copy number drivers of cancer, spatial tumor heterogeneity and spatial evolution | <https://github.com/raphael-group/CalicoST/> |
| **Inference of GRN** | SCING^65^ | Utilizes gradient boosting and mutual information to infer GRNs from scRNA-seq and spatial transcriptomics data | Effectively handles scRNA-seq and SRT data, producing robust GRN inference | Spatial transcriptomics data does not explicitly incorporate spatial information in the construction of gene regulatory networks | SCING infers robust GRNs, identifying cell type-specific genes and pathways underlying pathophysiology, while removing non-biological signals related to data quality, sample variability, and batch effects | https://github.com/XiaYangLabOrg/SCING |
|  | CLARIFY^66^ | Employs a multi-level graph autoencoder to model cellular networks, inferring cell-cell communication (CCC) and refining cell-specific GRNs | Refines cell-specific GRNs while modeling cellular networks | Strongly depends on accurate graph construction of complex cellular networks | As knowledge of the spatial landscape of GRNs increases, the CLARIFY model can be modified to incorporate new information | https://github.com/MihirBafna/CLARIFY |
|  | stKeep^67^ | Constructs a heterogeneous graph to capture complex relationships between cells/spots, genes, and cell states, using an attention-based multi-relation graph embedding algorithm for gene embeddings | Integrates multi-level information from genes, cells, and spatial data for precise GRN inference | Cannot identify novel gene-gene interactions or their directions | The learned cell-modules by incorporating information from related genes and histological regions, and semantically linked cells, facilitates detecting finer cell-states within TME. | https://github.com/cmzuo11/stKeep/ |

**Supplementary Table 3. Summary of spatial transcriptomics analysis methods.**

**References**

1 Wolf, F. A., Angerer, P. & Theis, F. J. SCANPY: large-scale single-cell gene expression data analysis. *Genome Biol* **19**, 15, doi:10.1186/s13059-017-1382-0 (2018).

2 Traag, V. A., Waltman, L. & van Eck, N. J. From Louvain to Leiden: guaranteeing well-connected communities. *Sci Rep* **9**, 5233, doi:10.1038/s41598-019-41695-z (2019).

3 Hu, J. *et al.* SpaGCN: Integrating gene expression, spatial location and histology to identify spatial domains and spatially variable genes by graph convolutional network. *Nat Methods* **18**, 1342-1351, doi:10.1038/s41592-021-01255-8 (2021).

4 Zhao, E. *et al.* Spatial transcriptomics at subspot resolution with BayesSpace. *Nat Biotechnol* **39**, 1375-1384, doi:10.1038/s41587-021-00935-2 (2021).

5 Pham, D. *et al.* Robust mapping of spatiotemporal trajectories and cell–cell interactions in healthy and diseased tissues. *Nat Commun* **14**, 7739 (2023).

6 Xu, H. *et al.* Unsupervised spatially embedded deep representation of spatial transcriptomics. *Genome Med* **16**, 12, doi:10.1186/s13073-024-01283-x (2024).

7 Li, J., Chen, S., Pan, X., Yuan, Y. & Shen, H. B. Cell clustering for spatial transcriptomics data with graph neural networks. *Nat Comput Sci* **2**, 399-408, doi:10.1038/s43588-022-00266-5 (2022).

8 Dong, K. & Zhang, S. Deciphering spatial domains from spatially resolved transcriptomics with an adaptive graph attention auto-encoder. *Nat Commun* **13**, 1739, doi:10.1038/s41467-022-29439-6 (2022).

9 Zuo, C. *et al.* Elucidating tumor heterogeneity from spatially resolved transcriptomics data by multi-view graph collaborative learning. *Nat Commun* **13**, 5962, doi:10.1038/s41467-022-33619-9 (2022).

10 Ren, H., Walker, B. L., Cang, Z. & Nie, Q. Identifying multicellular spatiotemporal organization of cells with SpaceFlow. *Nat Commun* **13**, 4076, doi:10.1038/s41467-022-31739-w (2022).

11 Zong, Y. e. a. conST: an interpretable multi-modal contrastive learning framework for spatial transcriptomics. *Preprint at bioRxiv*, doi:<https://doi.org/10.1101/2022.01.14.476408> (2022).

12 Liu, W. *et al.* Joint dimension reduction and clustering analysis of single-cell RNA-seq and spatial transcriptomics data. *Nucleic Acids Res* **50**, e72, doi:10.1093/nar/gkac219 (2022).

13 Zuo, C., Xia, J. & Chen, L. Dissecting tumor microenvironment from spatially resolved transcriptomics data by heterogeneous graph learning. *Nat Commun* **15**, 5057, doi:10.1038/s41467-024-49171-7 (2024).

14 Li, Z. & Zhou, X. BASS: multi-scale and multi-sample analysis enables accurate cell type clustering and spatial domain detection in spatial transcriptomic studies. *Genome Biol* **23**, 168, doi:10.1186/s13059-022-02734-7 (2022).

15 Long, Y. *et al.* Spatially informed clustering, integration, and deconvolution of spatial transcriptomics with GraphST. *Nat Commun* **14**, 1155, doi:10.1038/s41467-023-36796-3 (2023).

16 Edsgärd, D., Johnsson, P. & Sandberg, R. Identification of spatial expression trends in single-cell gene expression data. *Nat Methods* **15**, 339-342 (2018).

17 Svensson, V., Teichmann, S. A. & Stegle, O. SpatialDE: identification of spatially variable genes. *Nat Methods* **15**, 343-346 (2018).

18 Zhu, J., Sun, S. & Zhou, X. SPARK-X: non-parametric modeling enables scalable and robust detection of spatial expression patterns for large spatial transcriptomic studies. *Genome Biol* **22**, 184 (2021).

19 Hao, M., Hua, K. & Zhang, X. SOMDE: a scalable method for identifying spatially variable genes with self-organizing map. *Bioinformatics* **37**, 4392-4398 (2021).

20 DeTomaso, D. & Yosef, N. Hotspot identifies informative gene modules across modalities of single-cell genomics. *Cell systems* **12**, 446-456. e449 (2021).

21 Hu, J. *et al.* SpaGCN: Integrating gene expression, spatial location and histology to identify spatial domains and spatially variable genes by graph convolutional network. *Nat Methods* **18**, 1342-1351 (2021).

22 Zhang, C., Dong, K., Aihara, K., Chen, L. & Zhang, S. STAMarker: determining spatial domain-specific variable genes with saliency maps in deep learning. *Nucleic Acids Res* **51**, e103-e103 (2023).

23 Wang, J. *et al.* Dimension-agnostic and granularity-based spatially variable gene identification using BSP. *Nat Commun* **14**, 7367 (2023).

24 Liang, Y. *et al.* PROST: quantitative identification of spatially variable genes and domain detection in spatial transcriptomics. *Nat Commun* **15**, 600 (2024).

25 Andersson, A. *et al.* Single-cell and spatial transcriptomics enables probabilistic inference of cell type topography. *Communications Biology* **3**, doi:10.1038/s42003-020-01247-y (2020).

26 Song, Q. & Su, J. DSTG: deconvoluting spatial transcriptomics data through graph-based artificial intelligence. *Briefings in Bioinformatics* **22**, doi:10.1093/bib/bbaa414 (2021).

27 Elosua-Bayes, M., Nieto, P., Mereu, E., Gut, I. & Heyn, H. SPOTlight: seeded NMF regression to deconvolute spatial transcriptomics spots with single-cell transcriptomes. *Nucleic Acids Res* **49**, e50-e50 (2021).

28 Dong, R. & Yuan, G.-C. SpatialDWLS: accurate deconvolution of spatial transcriptomic data. *Genome Biology* **22**, doi:10.1186/s13059-021-02362-7 (2021).

29 Biancalani, T. *et al.* Deep learning and alignment of spatially resolved single-cell transcriptomes with Tangram. *Nature Methods* **18**, 1352-1362, doi:10.1038/s41592-021-01264-7 (2021).

30 Cable, D. M. *et al.* Robust decomposition of cell type mixtures in spatial transcriptomics. *Nature Biotechnology* **40**, 517-526, doi:10.1038/s41587-021-00830-w (2021).

31 Kleshchevnikov, V. *et al.* Cell2location maps fine-grained cell types in spatial transcriptomics. *Nature Biotechnology* **40**, 661-671, doi:10.1038/s41587-021-01139-4 (2022).

32 Lopez, R. *et al.* DestVI identifies continuums of cell types in spatial transcriptomics data. *Nature Biotechnology* **40**, 1360-1369, doi:10.1038/s41587-022-01272-8 (2022).

33 Miller, B. F., Huang, F., Atta, L., Sahoo, A. & Fan, J. Reference-free cell type deconvolution of multi-cellular pixel-resolution spatially resolved transcriptomics data. *Nature Communications* **13**, doi:10.1038/s41467-022-30033-z (2022).

34 Georgaka, S. *et al.* CellPie: a fast spatial transcriptomics topic discovery method via joint factorization of gene expression and imaging data. *bioRxiv*, 2023.2009. 2029.560213 (2023).

35 Thuilliez, C. *et al.* CellsFromSpace: a fast, accurate, and reference-free tool to deconvolve and annotate spatially distributed omics data. *Bioinformatics Advances* **4**, vbae081 (2024).

36 Chen, J. *et al.* Cell composition inference and identification of layer-specific spatial transcriptional profiles with POLARIS. *Science Advances* **9**, eadd9818 (2023).

37 Zhou, Z., Zhong, Y., Zhang, Z. & Ren, X. Spatial transcriptomics deconvolution at single-cell resolution using Redeconve. *Nat Commun* **14**, 7930 (2023).

38 Singh, R. *et al.* RETROFIT: Reference-free deconvolution of cell-type mixtures in spatial transcriptomics. *bioRxiv*, doi:10.1101/2023.06.07.544126 (2023).

39 Johansen, N., Hu, H. & Quon, G. Projecting RNA measurements onto single cell atlases to extract cell type-specific expression profiles using scProjection. *Nat Commun* **14**, 5192, doi:10.1038/s41467-023-40744-6 (2023).

40 Lu, Y., Chen, Q. M. & An, L. SPADE: spatial deconvolution for domain specific cell-type estimation. *Communications Biology* **7**, 469, doi:10.1038/s42003-024-06172-y (2024).

41 Ding, J. *et al.* SpatialCTD: a large-scale TME spatial transcriptomic dataset to evaluate cell type deconvolution for immuno-oncology. *bioRxiv*, 2023.2004. 2011.536333 (2023).

42 Swain, A. K., Pandit, V., Sharma, J. & Yadav, P. SpatialPrompt: spatially aware scalable and accurate tool for spot deconvolution and domain identification in spatial transcriptomics. *Communications Biology* **7**, 639 (2024).

43 Li, Y. & Luo, Y. Spatial transcriptomic cell-type deconvolution using graph neural networks. *bioRxiv* (2023).

44 Zhu, S. *et al.* STIE: Single-cell level deconvolution, convolution, and clustering in in situ capturing-based spatial transcriptomics. *Nat Commun* **15**, 7559 (2024).

45 Dries, R. *et al.* Giotto: a toolbox for integrative analysis and visualization of spatial expression data. *Genome Biology* **22**, doi:10.1186/s13059-021-02286-2 (2021).

46 Shao, X. *et al.* Knowledge-graph-based cell-cell communication inference for spatially resolved transcriptomic data with SpaTalk. *Nature Communications* **13**, doi:10.1038/s41467-022-32111-8 (2022).

47 Jin, S., Plikus, M. V. & Nie, Q. CellChat for systematic analysis of cell-cell communication from single-cell and spatially resolved transcriptomics. *BioRxiv*, 2023.2011. 2005.565674 (2023).

48 Li, Z., Wang, T., Liu, P. & Huang, Y. SpatialDM for rapid identification of spatially co-expressed ligand–receptor and revealing cell–cell communication patterns. *Nature Communications* **14**, doi:10.1038/s41467-023-39608-w (2023).

49 Yuan, Y. & Bar-Joseph, Z. GCNG: graph convolutional networks for inferring gene interaction from spatial transcriptomics data. *Genome Biology* **21**, doi:10.1186/s13059-020-02214-w (2020).

50 Li, H. *et al.* Decoding functional cell–cell communication events by multi-view graph learning on spatial transcriptomics. *Brief Bioinform* **24**, bbad359 (2023).

51 Li, R. & Yang, X. De novo reconstruction of cell interaction landscapes from single-cell spatial transcriptome data with DeepLinc. *Genome Biology* **23**, doi:10.1186/s13059-022-02692-0 (2022).

52 Fischer, D. S., Schaar, A. C. & Theis, F. J. Modeling intercellular communication in tissues using spatial graphs of cells. *Nature Biotechnology* **41**, 332-336, doi:10.1038/s41587-022-01467-z (2022).

53 Zuo, C., Xia, J. & Chen, L. Dissecting tumor microenvironment from spatially resolved transcriptomics data by heterogeneous graph learning. *Nature Communications* **15**, doi:10.1038/s41467-024-49171-7 (2024).

54 Cheng, J., Yan, L., Nie, Q. & Sun, X. Modeling and inference of spatial intercellular communications and multilayer signaling regulations using stMLnet. *BioRxiv*, 2022.2006. 2027.497696 (2022).

55 Tanevski, J., Flores, R. O. R., Gabor, A., Schapiro, D. & Saez-Rodriguez, J. Explainable multiview framework for dissecting spatial relationships from highly multiplexed data. *Genome Biology* **23**, doi:10.1186/s13059-022-02663-5 (2022).

56 Arnol, D., Schapiro, D., Bodenmiller, B., Saez-Rodriguez, J. & Stegle, O. Modeling Cell-Cell Interactions from Spatial Molecular Data with Spatial Variance Component Analysis. *Cell Reports* **29**, 202-211.e206, doi:10.1016/j.celrep.2019.08.077 (2019).

57 Zhu, J., Dai, H. & Chen, L. Revealing cell–cell communication pathways with their spatially coupled gene programs. *Briefings in Bioinformatics* **25**, doi:10.1093/bib/bbae202 (2024).

58 Cang, Z. *et al.* Screening cell–cell communication in spatial transcriptomics via collective optimal transport. *Nat Methods* **20**, 218-228, doi:10.1038/s41592-022-01728-4 (2023).

59 Abdelaal, T. *et al.* SIRV: Spatial inference of RNA velocity at the single-cell resolution. *NAR genomics and bioinformatics* **6**, lqae100 (2024).

60 Zuo, C. *et al.* Elucidating tumor heterogeneity from spatially resolved transcriptomics data by multi-view graph collaborative learning. *Nature Communications* **13**, doi:10.1038/s41467-022-33619-9 (2022).

61 Zhou, P., Bocci, F., Li, T. & Nie, Q. Spatial transition tensor of single cells. *Nature Methods* **21**, 1053-1062, doi:10.1038/s41592-024-02266-x (2024).

62 Shen, X. *et al.* Inferring cell trajectories of spatial transcriptomics via optimal transport analysis. *bioRxiv*, 2023.2009. 2004.556175 (2023).

63 Hou, W. & Ji, Z. Decomposing spatial heterogeneity of cell trajectories with Paella. *bioRxiv*, 2022.2009. 2005.506682 (2022).

64 Ma, C. *et al.* Inferring allele-specific copy number aberrations and tumor phylogeography from spatially resolved transcriptomics. *Nat Methods*, 1-9 (2024).

65 Littman, R., Cheng, M., Wang, N., Peng, C. & Yang, X. SCING: Inference of robust, interpretable gene regulatory networks from single cell and spatial transcriptomics. *Iscience* **26** (2023).

66 Bafna, M., Li, H. & Zhang, X. CLARIFY: cell–cell interaction and gene regulatory network refinement from spatially resolved transcriptomics. *Bioinformatics* **39**, i484-i493 (2023).

67 Zuo, C., Xia, J. & Chen, L. Dissecting tumor microenvironment from spatially resolved transcriptomics data by heterogeneous graph learning. *Nat Commun* **15**, 5057, doi:10.1038/s41467-024-49171-7 (2024).
